# Integrated Meta-omics Approaches To Understand the Microbiome of Spontaneous Fermentation of Traditional Chinese Pu-erh Tea

**DOI:** 10.1128/mSystems.00680-19

**Published:** 2019-11-19

**Authors:** Ming Zhao, Xiao Q. Su, Bo Nian, Li J. Chen, Dong L. Zhang, Shuang M. Duan, Li Y. Wang, Xing Y. Shi, Bin Jiang, Wei W. Jiang, Cai Y. Lv, Dao P. Wang, Yang Shi, Ying Xiao, Jian-Lin Wu, Ying H. Pan, Yan Ma

**Affiliations:** a College of Longrun Pu-erh Tea, Yunnan Agricultural University, Kunming, Yunnan, China; b State Key Laboratory of Conservation and Utilization of Bio-resources in Yunnan, Yunnan Agricultural University, Kunming, Yunnan, China; c Institute of Crop Sciences, Chinese Academy of Agricultural Sciences, Beijing, China; d State Key Laboratory of Quality Research in Chinese Medicine, Macau Institute for Applied Research in Medicine and Health, Macau University of Science and Technology, Macau, China; e The Key Laboratory of Medicinal Plant Biology of Yunnan Province, Yunnan Agricultural University, Kunming, Yunnan, China; f National & Local Joint Engineering Research Center on Germplasm Innovation & Utilization of Chinese Medicinal Materials in Southwestern China, Yunnan Agricultural University, Kunming, Yunnan, China; g Hangzhou Tea Research Institute, CHINA COOP, Hangzhou, China; University of California San Diego

**Keywords:** carbohydrate-active enzymes, food fermentation, meta-omics, microbiome, pu-erh tea, systemic view

## Abstract

The microbiome in fermentation has direct impacts on the quality of fermented foods and is of great scientific and commercial interest. Despite considerable effort to explain the microbial metabolism associated with food fermentation, the role of the microbiome in pu-erh tea fermentation remains unknown. Here, we applied integrated meta-omics approaches to characterize the microbiome in two repeated fermentations of pu-erh tea. Metabarcoding analysis of bacterial 16S rRNA genes showed a decrease in the proportion of *Proteobacteria* and an increase in the abundance of *Firmicutes* during fermentation. Metabarcoding analysis of fungal internal transcribed spacer (ITS) sequence demonstrated that *Rasamsonia*, *Thermomyces*, and *Aspergillus* were dominant at the intermediate stage, whereas *Aspergillus* was dominant at other stages in fermentation. Metaproteomics analysis assigned primary microbial metabolic activity to metabolism and identified microbial carbohydrate-active enzymes involved in the degradation of polysaccharides including cellulose, xylan, xyloglucan, pectin, starch, lignin, galactomannan, and chitin. Metabolomics and high-performance liquid chromatography analysis revealed that levels of phenolic compounds, including gallates, decreased whereas contents of gallic acid and ellagic acid significantly increased after fermentation (*P* < 0.05). The changes in levels of gallates and gallic acid were associated with the hydrolysis of tannase. Glycoside hydrolases, phenol 2-monooxygenase, salicylaldehyde dehydrogenase, salicylate 1-monooxygenase, catechol *O*-methyltransferase, catechol dioxygenase, and quercetin 2,3-dioxygenases were hypothesized to be related to oxidation, conversion, or degradation of phenolic compounds. We demonstrated microbiota in fermentation and their function in the production of enzymes related to the degradation of polysaccharides, and metabolism of phenolic compounds, resulting in changes in metabolite contents and the quality of pu-erh tea.

**IMPORTANCE** Fermented foods play important roles in diets worldwide and account for approximately one-third of all foods and beverages consumed. To date, traditional fermentation has used spontaneous fermentation. The microbiome in fermentation has direct impacts on the quality and safety of fermented foods and contributes to the preservation of traditional methods. Here, we used an integrated meta-omics approach to study the microbiome in the fermentation of pu-erh tea, which is a well-known Chinese fermented food with a special flavor and healthful benefits. This study advanced the knowledge of microbiota, metabolites, and enzymes in the fermentation of pu-erh tea. These novel insights shed light onto the complex microbiome in pu-erh fermentation and highlight the power of integrated meta-omics approaches in understanding the microbiome in food fermentation ecosystems.

## Abstract (precis)

Fermented foods play important roles in diets worldwide and account for approximately one-third of all foods and beverages consumed. To date, traditional fermentation has used spontaneous fermentation. The microbiome in fermentation has direct impacts on the quality and safety of fermented foods and contributes to the preservation of traditional methods. Here, we used an integrated meta-omics approach to study the microbiome in the fermentation of pu-erh tea, which is a well-known Chinese fermented food with a special flavor and healthful benefits. This study advanced the knowledge of microbiota, metabolites, and enzymes in the fermentation of pu-erh tea. These novel insights shed light onto the complex microbiome in pu-erh fermentation and highlight the power of integrated meta-omics approaches in understanding the microbiome in food fermentation ecosystems.

## INTRODUCTION

Fermentation is an ancient method of preserving food that depends on the reproducible formation of multiple species of microbial communities (1). It has been applied to food processing for thousands of years to extend shelf life and enhance nutritional properties (2). It remains a major technology for essential food production and can be used to produce healthy foods with a wide diversity of flavors, aromas, and textures, including alcoholic beverages, vinegar, pickled vegetables, sausage, cheese, yogurt, sauces and pastes, and bread (3). Fermented foods play important roles in diets worldwide and account for approximately one-third of all food and beverage consumption (4). To date, the traditional fermentation of foods such as cider, cereal-based beverages, kefir, and kombucha has used spontaneous fermentation (5). Complex and dynamic microbial ecosystems, in which bacteria, yeast, and filamentous fungi cohabit, interact, and communicate, are fundamental to the quality and safety of these fermented foods. The microbiome also contributes to the preservation of traditional methods (1, 6). In addition, food fermentation is an excellent model to investigate the microbial ecology of natural environments, owing to its simplicity, reproducibility, and accessibility and the cultivability and ease of manipulation of its associated microbial communities (1, 7). Therefore, the microbiome in food fermentation is of great scientific and commercial interest.

The field of microbiome biology (microbial community and functional analysis) has expanded enormously in the era of high-throughput functional genomics (8) and is beginning to incorporate multiple meta-omics approaches, including metagenomics, metatranscriptomics, metaproteomics, and metabolomics (9). Integration of these meta-omics approaches allows for the systematic study of microbial species, genes, metabolites, and activities; it contributes to our understanding of the organization, function, and interactions of organisms within communities (10, 11). Meta-omics approaches have shed light onto complex microbial communities that were once unknown (12), for example, microbiota in acid mine drainage (13), gastrointestinal microbiota (14), and microbiota in fermentation processes (15). Therefore, integration of meta-omics approaches is useful in the study of the microbiome in food fermentation.

Unlike the fermentation of the most widely consumed black tea, which is actually the oxidation of catechins, pu-erh tea is produced by a spontaneous microbial fermentation. As a well-known traditional Chinese fermented food with multiple health benefits, such as hypolipidemic, antimutagenic, antioxidative, antitumor, antiobesity, and toxicity-suppressing activities (16), it is popular in Southeast Asia and is becoming increasingly popular in the Western world. Microbial communities in the fermentation of pu-erh tea have previously been studied using culture methods and culture-independent approaches, including denaturing gradient gel electrophoresis, 16S rRNA gene clonal library, and next-generation sequencing (17). Recently, a study by Li et al. (18) using shotgun metagenomic and metabolomic analyses showed that significant variations in the composition of microbiota, collective functional genes, and flavor compounds occurred during the fermentation process. In a previous study, we developed an integrated metagenomics/metaproteomics approach and used the approach to investigate the microbial communities and enzymes of one fermented sample of pu-erh tea (19). However, to our knowledge, there have been no reports conducted using an integrated meta-omics approach to study the microbiome in pu-erh tea fermentation. In addition, little is known about the enzymes involved in the metabolism of phenolic compounds, which are essential for tea processing.

**FIG 1:**
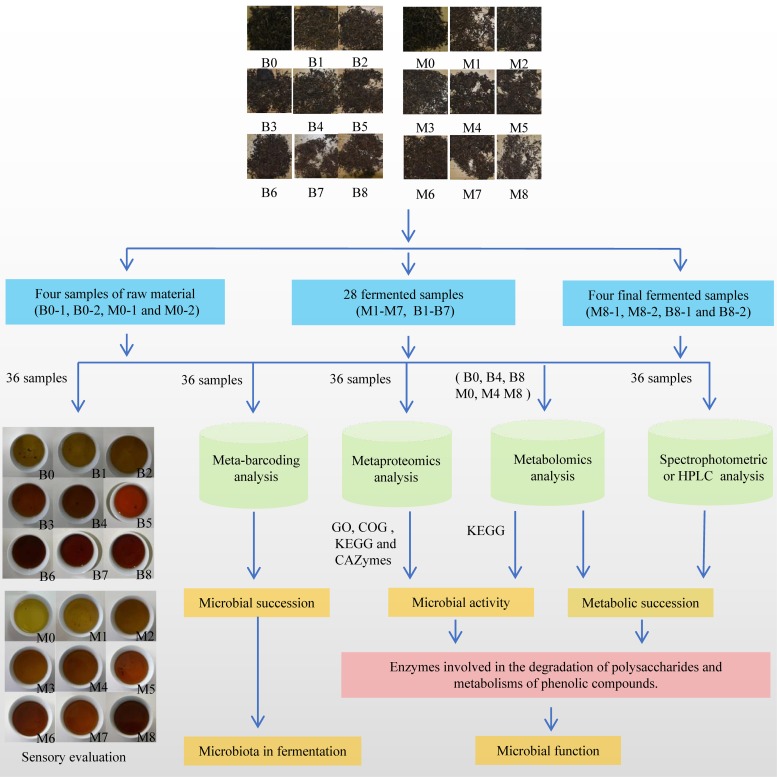
Outline of this integrated meta-omics study.

To conduct a systematic review of the fermentation mechanism of pu-erh tea, the microbiome in fermentation was studied using an integrated meta-omics approach. Bacterial 16S rRNA gene and fungal internal transcribed spacer 1 (ITS1) amplicon sequencing was used to characterize microbial succession during fermentation; the microbial activity was studied using a liquid chromatography-tandem mass spectrometry (LC-MS/MS)-based metaproteomics analysis. Metabolic succession was detected by high-performance liquid chromatography (HPLC) and an ultrahigh-performance liquid chromatography (UPLC)-MS/MS-based metabolomics analysis. Further, we surveyed microbial enzymes involved in the degradation of polysaccharides and the metabolism of phenolic compounds (Fig. 1). This work advanced the production of pu-erh tea and highlights the power of integrated meta-omics approaches in understanding the microbiome in food fermentation ecosystems.

## RESULTS AND DISCUSSION

### Fermentation of pu-erh tea and sample collection.

Two repeats of spontaneous fermentation of pu-erh tea (FB and FM) were developed based on the traditional method. In total, 36 samples were collected and analyzed, as outlined in Fig. 1 and Table 1. Sensory evaluation revealed that the infusion of raw tea material was slightly astringent in taste and yellow, but after fermentation, the tea infusion became mellow in taste and reddish-brown. This sensory quality change was similar to general fermentation observations of pu-erh tea. It is believed that changes in sensory qualities are closely related to changes in chemical metabolites and caused by microbial activity. Therefore, the composition and activity of microbes in the fermentation process were further investigated by integrated meta-omics approaches.

**TABLE 1:** Samples of tea leaves collected in fermentation of pu-erh tea and the designed analyses^*a*^

Sample	Collection date, day/mo (2014)	Sample identifier by repeat and sample			
		Repeat FB		Repeat FM	
		First sample	Second sample	First sample	Second sample
Raw material	10/10	FB0-1	FB0-2	FM0-1	FM0-2
Repiling					
First	18/10	FB1-1	FB1-2	FM1-1	FM1-2
Second	25/10	FB2-1	FB2-2	FM2-1	FM2-2
Third	30/10	FB3-1	FB3-2	FM3-1	FM3-2
Fourth	10/11	FB4-1	FB4-2	FM4-1	FM4-2
Fifth	20/11	FB5-1	FB5-2	FM5-1	FM5-2
Sixth	26/11	FB6-1	FB6-2	FM6-1	FM6-2
Seventh	29/11	FB7-1	FB7-2	FM7-1	FM7-2
Final fermented tea leaves	1/12	FB8-1	FB8-2	FM8-1	FM8-2

a All samples were analyzed by sensory evaluation, HPLC, the spectrophotometric method, metabarcoding analysis, and metaproteomics analysis. Additionally, raw material, the fourth repiling, and the final fermented tea leaves were subjected to metabolomics analysis.

### Microbiota in the fermentation of pu-erh tea.

**FIG 2:**
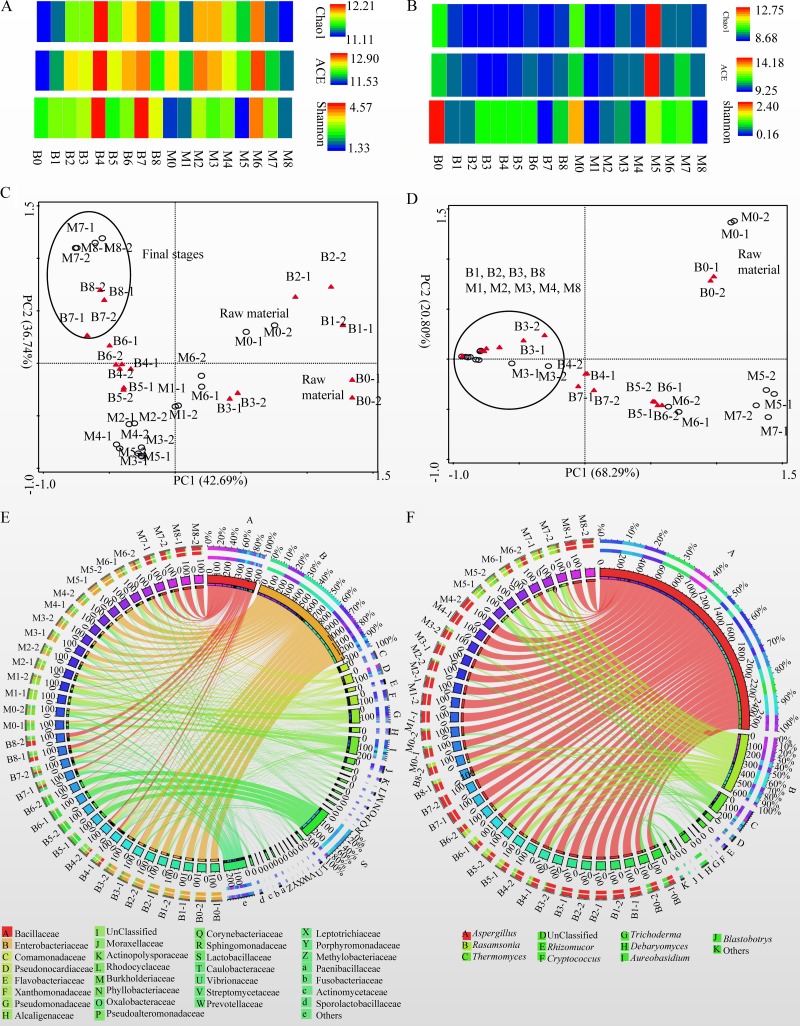
Microbiota in the fermentation of pu-erh tea. (A and B) Bacterial (A) and fungal (B) Chao1 and abundance-based coverage estimates (ACE) and Shannon indices. (C and D) PCA of relative abundance (RA) of bacterial (C) and fungal (D) OTUs in each sample. (E and F) Circos plots of changes of major bacterial families (E) and fungal genera (F) (RAs, >1% of the total sequences) in fermentation. “Others” are composed of the family or genus showing a percentage of reads of <1.0% of the total reads in each sample.

A total of 877,307 bacterial 16S rRNA and 1,740,425 fungal ITS sequences were obtained from 36 samples of tea leaves, and most of the microbial diversity was captured by this analysis (see Fig. S1A and B in the supplemental material). As the fermentation process progresses, bacterial richness and diversity increase, showing a peak in B4 or M6 and decreasing slightly in the last stages of fermentation (Fig. 2A). Fungal richness and diversity decreased in the initial stage of fermentation, and increased slightly during the middle and final stages of fermentation (Fig. 2B).

10.1128/mSystems.00680-19.2 FIG S1 (A and B) Rarefaction curves of bacterial 16S rRNA genes (A) and fungal ITS sequences (B). (C) Changes in RAs of bacterial phyla in fermentation. (D) Changes in RAs of fungal phyla in fermentation. (E) Changes in RAs of major bacterial families (>1% of the total sequences) in fermentation. (F) Changes in RAs of major fungal genera (>1% of the total sequences) in fermentation. “Others” are composed of the family or genus showing a percentage of reads of <1.0% of the total reads in each sample. Download FIG S1, TIF file, 7.3 MB. Copyright © 2019 Zhao et al. 2019 Zhao et al. This content is distributed under the terms of the Creative Commons Attribution 4.0 International license.

Principal-component analysis (PCA) based on the relative abundance (RA) of operational taxonomic units (OTUs) revealed that both bacterial and fungal communities in the raw material (B0 and M0) differed from those of other fermented stages (Fig. 2C and D); bacterial communities in the final stages of the fermentation of tea leaves (B7, B8, M7, and M8) were similar to each other and yet they differed from those of other stages. However, fungal communities in the final fermentation steps of tea leaves (B8 and M8) were similar to those in other fermented samples (B1, B2, B3, M1, M2, M3, and M4).

Bacterial OTUs were classified into 31 phyla (Data Set S1, sheet 1). At the phylum level, there was a decrease in the proportion of *Proteobacteria* and an increase in the abundance of *Firmicutes*. Additionally, the RA of *Actinobacteria* increased to 15.37% (B4), 24.90% (B8), and 57.51% (M7) (Fig. S1C). More than 80% of bacterial OTUs were assigned to 201 families (Data Set S1, sheet 2). As the fermentation process progressed, proportions of *Enterobacteriaceae* decreased in FB, whereas they increased in FM. The abundance of *Bacillaceae* increased at the final stages in both FB and FM with RAs of 44.92% (B8) and 89.05% (M8). Additionally, RAs of *Lactobacillaceae* were greater than 70% in B5 and B6, and the RA of *Pseudomonadaceae* was 46.56% in M1 (Fig. 2E and Fig. S1E). Similarly to our previous findings (20), the RA of *Enterobacteriaceae* decreased and the proportions of *Bacillaceae* increased during the fermentation process of pu-erh tea.

10.1128/mSystems.00680-19.7 DATA SET S1 The microbial community in fermented tea samples. Download Data Set S1, XLSX file, 1.6 MB. Copyright © 2019 Zhao et al. 2019 Zhao et al. This content is distributed under the terms of the Creative Commons Attribution 4.0 International license.

Fungal OTUs were classified into four phyla and unclassified fungi. The dominant phylum was Ascomycota, which accounted for 84.76% to 99.94% of the total fungi (Fig. S1D and Data Set S1, sheet 3). More than 87% of fungal reads were classified into 166 genera; *Aspergillus* and *Debaryomyces* were dominant genera in B0 with RAs of 55.69% and 28.90%, respectively, whereas the RA of *Aspergillus* was 80.03% in M0. In combination with *Aspergillus* (RAs of 10.05 to 63.78%), *Rasamsonia* (19.58 to 58.68%) and *Thermomyces* (3.61 to 18.7%) were the dominant fungi in the samples of B4, B5, B6, M5, M6, and M7, whereas *Aspergillus* was the dominant fungus in other fermented samples with RAs greater than 65% (Fig. 2F, Fig. S1F, and Data Set S1, sheet 4). The dominance of *Aspergillus* and the presence of *Rasamsonia* and *Thermomyces* in the fermentation of pu-erh tea have been previously reported (19, 21).

**FIG 3:**
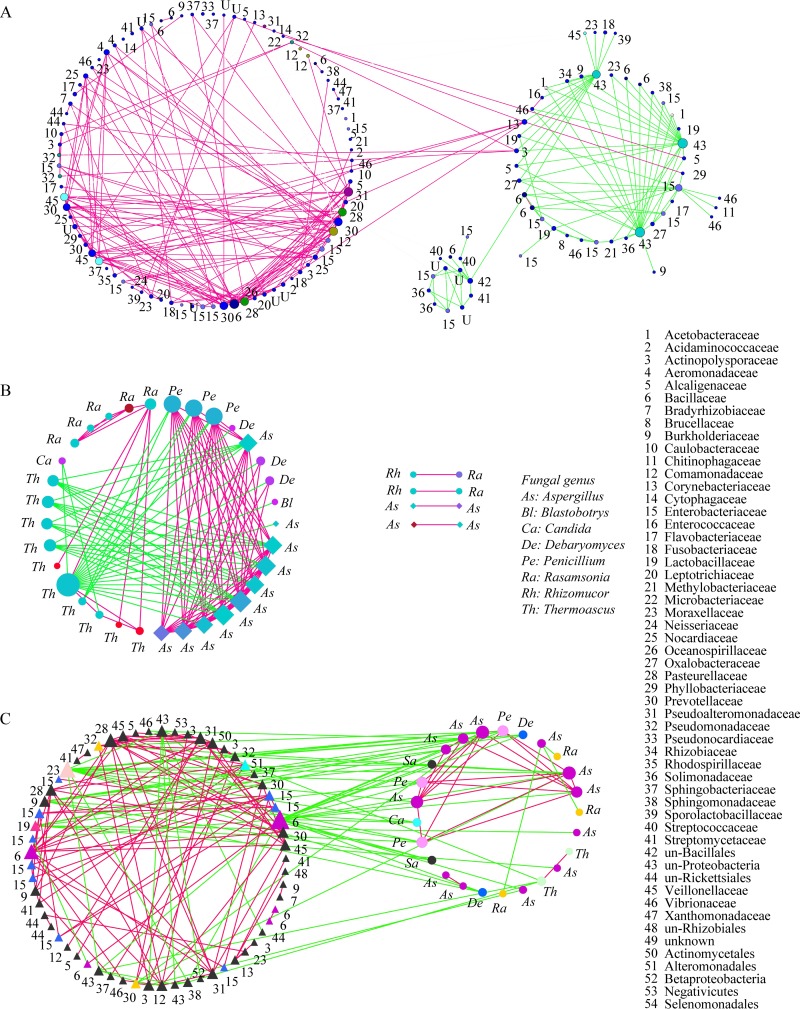
Cooccurrence networks of OTUs. (A) Bacterium-bacterium cooccurrence network. (B) Fungus-fungus cooccurrence network. (C) Bacterium-fungus cooccurrence network. Nodes correspond to OTUs, and connecting edges indicate negative (green) or positive (red) correlations between them.

To obtain a measure of microbial association, three OTU cooccurrence networks were constructed. In the bacterial cooccurrence network, most of the OTUs corresponding to *Bacillaceae*, *Comamonadaceae*, *Pseudoalteromonadaceae*, *Pseudomonadaceae*, *Phyllobacteriaceae*, and *Vibrionaceae* cooccurred with others in fermentation. OTUs assigned to unclassified groups in *Rickettsiales* and *Enterobacteriaceae* were negatively correlated with other bacteria (Fig. 3A). In the fungal network, most of the OTUs assigned to *Thermomyces* and *Thermoascus* have negative correlations with other genera, whereas the OTUs corresponding to *Aspergillus*, *Rasamsonia*, *Penicillium*, and *Debaryomyces* cooccurred with each other (Fig. 3B). The network of OTUs of 16S RNA genes and ITS sequences showed that bacteria and fungi were mutually exclusive in fermentation. For example, members of the *Bacillaceae*, *Pseudoalteromonadaceae*, *Comamonadaceae*, and *Enterobacteriaceae* showed negative correlations with fungi in *Aspergillus*, *Rasamsonia*, *Penicillium*, *Debaryomyces*, and *Saccharomycetes* (Fig. 3C).

### Overview of metaproteomics results.

After protein extraction, LC-MS/MS analyses, and a database search, 68 to 1,582 microbial proteins in each repeated analysis and 4,623 and 6,505 unique proteins in FB and FM, respectively, were identified and further annotated, with proteins identified as malate dehydrogenase, superoxide dismutase, and catalase, among others (Data Set S2, sheets 1 and 2). In the Gene Ontology (GO) annotation, the majority of identified proteins categorized as molecular functions were primarily catalytic activity and binding; those categorized as biological process were cellular process and metabolic process; the proteins categorized as cellular components were cell parts and protein-containing complex. The majority of enzyme classes were oxidoreductases, transferases, and hydrolases (Fig. S2A and Data Set S2, sheets 3 and 4). The major categories identified by the Cluster of Orthologous Group analysis were energy production and conversion, translation, ribosomal structure and biogenesis, posttranslational modification, protein turnover and chaperones, amino acid transport and metabolism, and carbohydrate transport and metabolism (Fig. S2B and Data Set S2, sheet 5). A total of 116 KEGG pathways were annotated, with the most common pathways identified as glycolysis/gluconeogenesis (ko00010), ribosome (ko03008), oxidative phosphorylation (ko00190), and citrate cycle (ko00020) (Fig. S2C and Data Set S2, sheet 6). These KEGG pathways grouped into cellular processes, environmental information processing, genetic information processing, and metabolism. KEGG pathways in metabolism were further associated with classes of amino acid metabolism, biosynthesis of other secondary metabolites, and carbohydrate metabolism (Fig. S2C). Overall, the majority of identified proteins were assigned to cellular process and metabolic process in the GO analysis and enriched in pathways belonging to metabolism or genetic information processing. These data support the findings from microbial growth and reproduction.

10.1128/mSystems.00680-19.3 FIG S2 Results of the functional analysis of metaproteins. (A) Gene Ontology (GO) and Enzyme Class (EC) analyses of unique proteins. (B) Clusters of Orthologous Groups of unique proteins (COG). (C) KEGG pathway annotation analysis of proteins and metabolites. Download FIG S2, TIF file, 9.0 MB. Copyright © 2019 Zhao et al. 2019 Zhao et al. This content is distributed under the terms of the Creative Commons Attribution 4.0 International license.

10.1128/mSystems.00680-19.8 DATA SET S2 Results of the metaproteomics analysis. Download Data Set S2, XLSX file, 12.4 MB. Copyright © 2019 Zhao et al. 2019 Zhao et al. This content is distributed under the terms of the Creative Commons Attribution 4.0 International license.

### Metabolic succession in fermentation.

**FIG 4:**
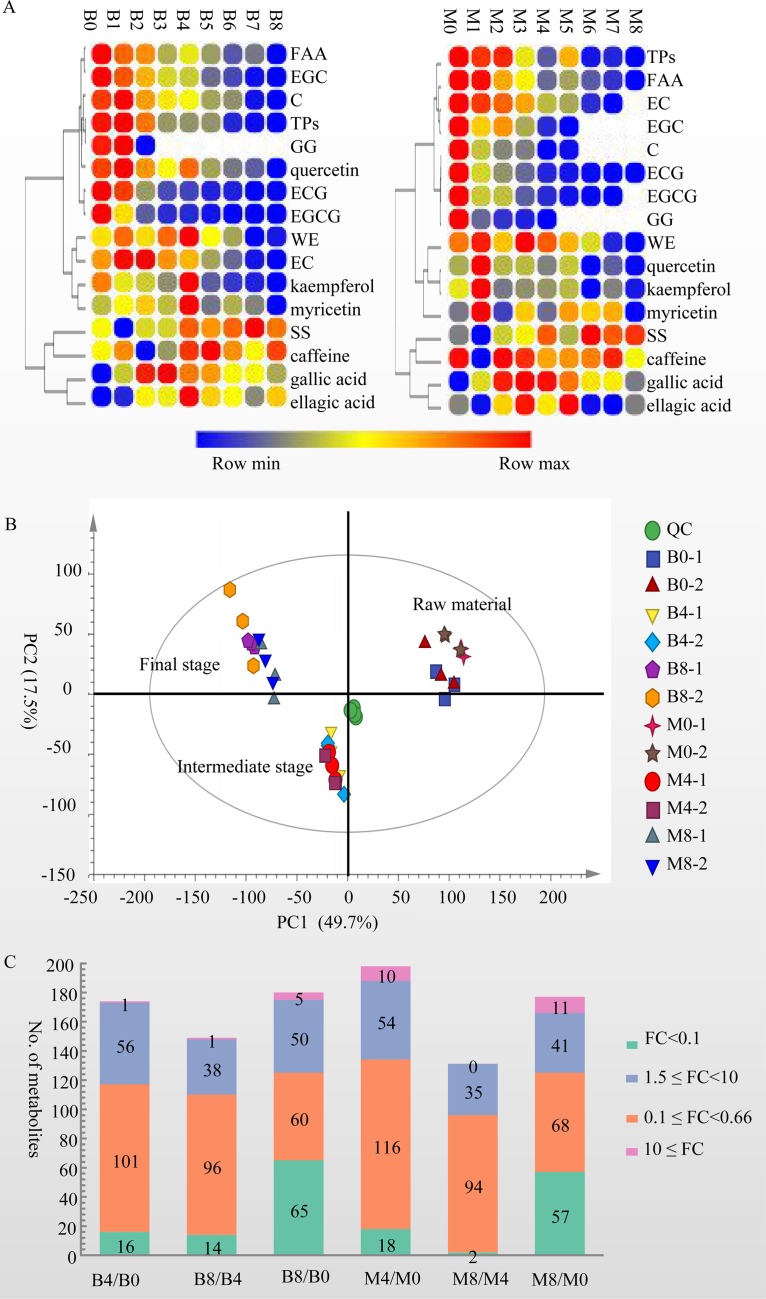
Results of metabolomic analysis. (A) Change trend for compounds measured by spectrophotometric and HPLC methods. (B) Principal-component analysis (PCA) of the items detected by positive and negative data in the metabolomic analysis. (C) Distribution of fold changes (FCs) of metabolites.

The concentrations of 16 characteristic components of tea were measured by HPLC or spectrophotometric methods. We observed three change trends among the results (Fig. 4A; Table 2): (i) the levels of tea polyphenols (TPs), free amino acids (FAA), (−)-epigallocatechin (EGC), (+)-catechin (C), 1,4,6-tri-*O-*galloyl-*β*-d-glucose (GG), (−)-epicatechin 3-*O*-gallate (ECG), and (−)-epigallocatechin 3-*O*-gallate (EGCG) decreased with the development of fermentation; (ii) the contents of water extractions (WE), kaempferol, quercetin, myricetin, and (−)-epicatechin (EC) increased at the initial stage and then decreased significantly after fermentation (*P* < 0.05); and (iii) the levels of soluble sugar (SS), gallic acid, and ellagic acid increased significantly after fermentation (*P* < 0.05). Additionally, the caffeine content increased significantly (*P* < 0.05) in FB but showed no significant change in FM (*P* > 0.05).

**TABLE 2:** Contents of chemical compounds measured by spectrophotometric and HPLC methods

Sample	Content, mean ± SD (*n* = 6)^*a*^															
	%				Concn (mg/g)											
	WEs	TPs	FAA	SS	C	EC	EGC	ECG	EGCG	GG	Gallic acid	Caffeine	Quercetin	Kaempferol	Myricetin	Ellagic acid
B0	51.70 ± 0.66 A	33.54 ± 0.13 A	1.76 ± 0.01 A	7.94 ± 0.03 A	6.53 ± 0.11 B	18.45 ± 0.15 B	28.28 ± 0.87 A	39.94 ± 2.59 A	47.30 ± 2.42 A	1.77 ± 0.69 A	1.1 ± 0.02 I	27.06 ± 0.33 CD	3.37 ± 0.31 A	1.43 ± 0.12 AB	0.45 ± 0.05 BC	17.01 ± 1.64 C
B1	55.21 ± 0.04 B	34.19 ± 0.08 A	1.54 ± 0.02 B	7.49 ± 0.04 B	7.32 ± 0.18 A	25.41 ± 0.69 A	23.68 ± 0.46 B	35.22 ± 2.66 B	26.71 ± 1.84 B	1.84 ± 0.10 A	14.8 ± 0.43 G	27.64 ± 0.43 BC	3.57 ± 0.67 A	1.15 ± 0.22 BC	0.50 ± 0.17 B	17.53 ± 2.94 C
B2	51.85 ± 1.25 A	29.45 ± 2.25 B	1.45 ± 0.00 C	7.880 ± 0.14 C	5.12 ± 0.11 C	25.75 ± 0.45 A	18.57 ± 0.27 C	12.18 ± 1.27 C	8.76 ± 0.77 C	0.51 ± 0.03 B	34.08 ± 0.10 B	25.64 ± 0.10 E	3.12 ± 0.16 AB	1.10 ± 0.03BC	0.53 ± 0.20 B	20.79 ± 2.60 B
B3	55.27 ± 0.95 B	19.49 ± 1.18 C	1.03 ± 0.05 D	7.87 ± 0.12 C	4.08 ± 0.08 D	18.65 ± 0.28 B	12.11 ± 0.19 D	6.02 ± 1.31 D	4.16 ± 0.29 D		36.07 ± 0.43 A	26.46 ± 0.24 D	2.79 ± 0.23 BC	0.99 ± 0.09 CD	0.45 ± 0.07 BC	20.35 ± 1.46 B
B4	58.31 ± 0.70 C	19.32 ± 1.41 C	1.15 ± 0.03 E	8.24 ± 0.14 A	3.64 ± 0.05 D	16.92 ± 0.17 C	11.44 ± 0.12 D	5.36 ± 0.96 D	3.86 ± 0.20 D		27.85 ± 0.2 C	28.10 ± 0.23 AB	3.26 ± 0.52 A	1.66 ± 0.76 A	0.67 ± 0.16 A	24.23 ± 3.34 A
B5	50.76 ± 0.95 D	19.31 ± 0.45 C	0.99 ± 0.00 D	8.14 ± 0.12 A	2.49 ± 0.06 E	8.99 ± 0.19 D	6.26 ± 0.16 E	2.80 ± 0.23 E	1.81 ± 0.12 E		25.46 ± 0.36 D	28.38 ± 0.29 A	2.52 ± 0.29 CD	0.90 ± 0.12 CD	0.40 ± 0.06 BC	21.30 ± 2.22 B
B6	48.15 ± 0.30 E	14.81 ± 0.42 D	0.84 ± 0.02 F	8.23 ± 0.04 A	2.21 ± 0.21 E	6.02 ± 0.30 E	4.45 ± 0.22 F	2.53 ± 2.17 EF	0.30 ± 0.60 EF		18.99 ± 0.28 E	27.60 ± 0.29 BC	2.39 ± 0.29 CDE	0.83 ± 0.13 CD	0.44 ± 0.18 BC	20.64 ± 0.70 B
B7	43.27 ± 0.45 F	13.69 ± 0.52 D	0.91 ± 0.00 G	8.41 ± 0.15 A	0.43 ± 0.86 F	2.02 ± 0.57 F	1.13 ± 2.26 G	0.12 ± 0.24 F	0.08 ± 0.15 F		18.16 ± 0.53 F	27.05 ± 0.95 CD	2.31 ± 0.26 DE	0.79 ± 0.08 CD	0.41 ± 0.07 BC	18.97 ± 1.95 BC
B8	43.94 ± 0.02 F	13.25 ± 0.37 D	0.65 ± 0.00 H	8.20 ± 0.01 A	0.19 ± 0.39 F	1.02 ± 0.01 G	0.44 ± 0.88 G	0.48 ± 0.32 EF	0.08 ± 0.15 F		12.79 ± 0.22 H	28.02 ± 0.25 AB	2.03 ± 0.34 E	0.73 ± 0.13 DE	0.32 ± 0.07 C	21.39 ± 1.67 B
M0	52.56 ± 0.92 A	29.34 ± 0.30 A	2.00 ± 0.02 A	7.94 ± 0.03 A	6.46 ± 0.39 A	17.76 ± 0.61 A	28.08 ± 2.04 A	37.81 ± 2.56 A	46.02 ± 2.81 A	2.36 ± 0.18 A	1.13 ± 0.06 E	25.74 ± 0.71 A	2.72 ± 0.35 BC	2.22 ± 0.27 B	0.59 ± 0.09 C	10.14 ± 1.07 C
M1	54.92 ± 0.56 B	27.80 ± 0.64 A	1.98 ± 0.09 A	7.84 ± 0.02 B	3.48 ± 0.72 B	15.92 ± 3.13 B	19.34 ± 4.15 B	15.88 ± 3.08 B	18.41 ± 3.64 B	0.76 ± 0.16 B	11.06 ± 2.10 C	18.56 ± 3.53 B	5.34 ± 0.37 A	4.32 ± 0.68 A	1.67 ± 0.28 A	7.02 ± 0.82 D
M2	50.50 ± 0.27 C	28.26 ± 0.95 A	1.60 ± 0.00 B	8.00 ± 0.02 A	2.91 ± 0.03 C	14.45 ± 0.19 B	21.76 ± 0.22 B	11.08 ± 0.85 C	17.52 ± 1.24 B	0.56 ± 0.05 C	22.84 ± 0.15 AB	25.88 ± 0.16 A	2.80 ± 0.18 B	1.40 ± 0.08 A	0.44 ± 0.04 BCD	14.50 ± 0.71 B
M3	55.73 ± 0.27 D	20.88 ± 0.00 B	1.42 ± 0.00 C	8.05 ± 0.08 A	2.81 ± 0.03 C	12.28 ± 0.12 C	13.64 ± 0.17 C	8.00 ± 0.49 D	8.72 ± 0.48 C	0.48 ± 0.07 CD	24.21 ± 0.15 A	25.22 ± 0.20 A	2.84 ± 0.19 B	1.57 ± 0.50 C	1.11 ± 0.37 B	19.42 ± 1.14 A
M4	53.36 ± 0.35 A	16.63 ± 2.61 B	1.04 ± 0.02 D	8.17 ± 0.01 C	1.63 ± 0.07 D	7.03 ± 0.28 D	8.21 ± 0.34 D	2.94 ± 0.36 E	4.08 ± 0.44 D	0.36 ± 0.04 D	23.65 ± 0.69 A	23.52 ± 0.78 A	2.39 ± 0.12 BC	1.62 ± 0.06 C	0.56 ± 0.03 C	13.88 ± 0.69 B
M5	50.91 ± 1.31 A	23.90 ± 0.28 B	1.18 ± 0.00 E	7.98 ± 0.02 A	1.76 ± 0.02 D	6.39 ± 0.06 D	6.37 ± 0.07 D	1.87 ± 0.05 EF	2.07 ± 0.06 DE		18.93 ± 0.17 B	23.74 ± 0.31 A	2.92 ± 1.21 B	1.87 ± 0.95 BC	1.24 ± 0.39 B	19.95 ± 1.10 A
M6	47.93 ± 1.87 E	14.51 ± 0.92 C	1.00 ± 0.00 D	8.24 ± 0.05 D		2.28 ± 0.13 E		0.95 ± 0.11 EF	1.04 ± 0.26 E		13.79 ± 0.57 C	24.05 ± 1.20 A	1.39 ± 0.39 D	0.49 ± 0.15 D	1.10 ± 0.31 B	6.96 ± 1.75 D
M7	43.07 ± 0.65 F	15.01 ± 0.60 D	0.91 ± 0.01 F	8.17 ± 0.05 D		0.82 ± 0.55 EF		0.60 ± 0.40 F	0.43 ± 0.50 E		13.49 ± 0.10 C	25.37 ± 0.36 A	2.12 ± 0.31 C	1.46 ± 0.22 C	1.19 ± 0.18 B	6.67 ± 0.92 D
M8	42.15 ± 0.01 F	13.88 ± 0.19 C	0.79 ± 0.02 G	8.20 ± 0.01 D				0.31 ± 0.36 F			6.71 ± 7.74 D	22.10 ± 5.66 A	1.45 ± 0.55 D	0.66 ± 0.17 D	0.26 ± 0.08 D	9.95 ± 1.65 C

a The different uppercase letters indicate significant differences among the values (*P* < 0.05).

A total of 11,423 *m/z* were detected in the metabolomics analysis (Data Set S3, sheets 1 and 2), and the PCA with 67.0% variation showed that the metabolites identified in samples from raw material (B0-1, B0-2, M0-1, and M0-2), the middle stage of fermentation (B4-1, B4-2, M4-1, and M4-2), and the final stage (B8-1, B8-2, M8-1, and M8-2) of fermentation were distinct (Fig. 4B). Two hundred ninety-eight metabolites for which relative levels changed significantly in comparisons of B4 and B0, B8 and B4, B8 and B0, M4 and M0, M8 and M4, and M8 and M0 (variable importance in projection [VIP] > 1.0, *P* < 0.05, and fold change [FC] > 1.5 or FC < 0.66) were identified, for example, rutin, catechin 3′-*O*-gallate, theogallin, and (±)-catechin. The majority of these metabolites belong to classes of flavonoids (117 metabolites), glycerophospholipids (56 metabolites), fatty acyls (18 metabolites), carboxylic acids and derivatives (17 metabolites), and organo-oxygen compounds (15 metabolites) (Data Set S3, sheets 2 and 3). Among the metabolites, 44 were annotated into 61 KEGG pathways, with highly represented pathways of flavonoid biosynthesis (ko00941), flavone and flavonol biosynthesis (ko00944), and biosynthesis of phenylpropanoids (ko01061), which included eight, five, and five metabolites, respectively. Twenty-eight KEGG pathway terms were annotated by both metaproteomics and metabolomics, including citrate cycle (ko00020) and glycerophospholipid metabolism (ko00564) (Data Set S3, sheet 4).

10.1128/mSystems.00680-19.9 DATA SET S3 Results of the metabolomics analysis. Download Data Set S3, XLSX file, 6.2 MB. Copyright © 2019 Zhao et al. 2019 Zhao et al. This content is distributed under the terms of the Creative Commons Attribution 4.0 International license.

Relative levels of 96 to 134 or 35 to 64 metabolites were decreased (VIP > 1.0, *P* < 0.05, and FC < 0.66) or increased significantly (VIP > 1.0, *P* < 0.05, and FC > 1.5) in comparison with B4/B0, B8/B4, B8/B0, M4/M0, M8/M4, and M8/M0, respectively (Fig. 4C). After fermentation, the relative levels of 124 and 125 metabolites were decreased significantly in comparisons of B8 and B0 and of M8 and M0, respectively. Among them, relative peak areas of 64 and 57 metabolites, respectively, decreased by more than 10-fold, including EGCG, theaflavin digallate, luteoliflavan, and l-theanine, whereas the relative peak areas of 55 and 52 metabolites significantly increased after fermentation in comparison of B8 and B0 and of M8 and M0, respectively, including margrapine A, kukoamine A, Thr-Trp-OH, Phe-Lys-OH, uridine, ellagic acid, and gallic acid (Data Set S3, sheet 5). HPLC determination verified the increasing contents of gallic acid, which increased 11.63 and 5.94 times in FB and FM, respectively.

### CAZyme analysis.

Carbohydrate-active enzymes (CAZymes) are involved in the assembly and breakdown of complex carbohydrates, including oligosaccharides or polysaccharides as well as glycoconjugates to nucleic acids, proteins, lipids, polyphenols, and other natural compounds (22). They are responsible for the synthesis (through glycosyltransferases [GTs]), degradation (glycoside hydrolases [GHs], polysaccharide lyases [PLs], carbohydrate esterases [CEs], and auxiliary activities [AAs]) and recognition (carbohydrate binding module [CBM]) of all the carbohydrates on Earth (23). Through dbCAN annotation, 284 and 471 proteins in FB and FM, respectively, and a total of 558 unique proteins were annotated to 131 items in the CAZy database; the identified proteins were distributed into all six families of GHs, GTs, AAs, CEs, PLs, and CBMs. Highly represented family items were AA3 (28 proteins), AA7 (20 proteins), AA2 (15 proteins), CBM (12 proteins), CE10 (17 proteins), GH13 (21 proteins), GH28 (18 proteins), GH3 (19 proteins), GH6 (23 proteins), GH72 (16 proteins), GT30 (17 proteins), GT4 (40 proteins), and PL1_4 (6 proteins) (Fig. S3A and Data Set S2, sheet 7).

10.1128/mSystems.00680-19.4 FIG S3 Results of the analysis of carbohydrate-active enzymes (CAZymes). (A) Overview of CAZyme families identified in pu-erh tea fermentation. (B) CAZymes involved in the degradation of polysaccharides. Download FIG S3, TIF file, 18.5 MB. Copyright © 2019 Zhao et al. 2019 Zhao et al. This content is distributed under the terms of the Creative Commons Attribution 4.0 International license.

In these CAZymes, 82 and 137 enzymes belonged to 31 and 39 families or subfamilies of CAZymes, respectively, which were hypothesized to degrade plant and fungal polysaccharides, including cellulose, xylan, xyloglucan, pectin, starch, lignin, galactomannan, and chitin (Fig. S3B and Data Set S2, sheet 8). For example, glucanase (GH5, -12, -16, -7, and -55; AA9 and AA11), cellobiohydrolase (GH7), glucosidase (GH1, -3, -17, and -55), and glucose-methanol-choline (GMC) oxidoreductase (AA3) may be involved in the biodegradation of cellulose. Peroxidase, aryl-alcohol dehydrogenase, GMC oxidoreductase, choline oxidase, and alcohol oxidase, belonging to AA2 or AA3, are involved in the degradation of lignin. A series of enzymes, including alpha-l-arabinofuranosidase (GH51, -54, and -62), alpha-l-rhamnosidase (GH78), alpha-*n*-arabinofuranosidase (GH51 and -54), beta-galactosidase (GH35 and -42), galactanase (GH53), endopolygalacturonase (GH28), exo-*a*-l-1,5-arabinanase (GH93), pectate lyase (PL1_7 and -3_2), pectin lyase (PL1_4), polygalacturonase (GH28), rhamnogalacturan acetylesterase (CE12), rhamnogalacturonase (GH28), and 1,4-beta-xylosidase (GH3), are able to degrade the pectin backbone and the side chains of the hairy regions of pectin. Galactosidase (GH35, -42, -27, and -36), arabinofuranosidase (GH51, -54, and -62), xylosidase (GH3), xylanase (GH10, -11, and -43), and alpha-l-fucosidase (GH29) are able to degrade the xylan and xyloglucan. Alpha-glucosidase (GH13 and -31) and alpha-amylase (GH13) can biodegrade starch. Galactosidase (GH27, -35, -36, and -42), glucosidase (GH1, -3, -17, and -55), beta-mannanase (GH5), and beta-mannosidase (GH2) degrade galactomannan. Chitinase (GH18), glucanase (GH5_9, -16, and -17), glucanosyltransferase (GH16, -17, and -72), glucan-1,3-beta-glucosidase (GH17), and beta-hexosaminidase (GH20) are involved in the degradation of chitin. Therefore, the microbiota involved in fermentation produce CAZymes related to the degradation of plant or fungal biomass polymers and use the resulting monomers and oligomers for their growth and reproduction (Fig. S3B and Data Set S2, sheet 8). Degradation of plant polysaccharides can destroy the cells of tea leaves. This is supported by the observation of the softening of tea leaves during the fermentation of pu-erh tea. Similarly, Wang et al. showed that the surfaces of tea leaves were covered by microbiota and the cell structures were largely disrupted after fermentation (24).

In the metabolomics analysis, 76 glycosides were identified, with the majority being glucosides (31 metabolites), rhamnosides (11 metabolites), glucuronides (nine metabolites), and glucopyranosides (seven metabolites). After fermentation, relative levels of 36 and 33 glycosides were decreased (VIP > 1.0, *P* < 0.05, and FC < 0.66), whereas six and five of them showed increased levels (VIP > 1.0, *P* < 0.05, and FC > 1.5) in B8/B0 and M8/M0, respectively (Data Set S3, sheet 6). GHs (EC 3.2.1.−) (25) and GTs (26) (EC 2.4.x.y) catalyze the hydrolysis and formation of the glycosidic bond; thus, they may be involved in the degradation or synthesis of glycosides. For example, glucosidase (e.g., A0A2G7G438 and A0A1F2K440), beta-glucuronidase (A0A100IUA9), and 1,3-beta-glucanosyltransferase (A0A0F4YK02) were suggested to be involved in the metabolism of glucosides and glucopyranosides. Galactopyranoside and galactoside were hydrolyzed by beta-galactosidases (A0A0F4Z133, A0A0L1J957, A0A1J6WUZ7, and A0A1L9RJP3). Alpha-l-rhamnosidase (A0A0R1I3B8) was suggested to hydrolyze rhamnoside (Table S1).

10.1128/mSystems.00680-19.5 TABLE S1 Enzymes involved in the hydrolysis of glycosides. Download Table S1, DOCX file, 0.03 MB. Copyright © 2019 Zhao et al. 2019 Zhao et al. This content is distributed under the terms of the Creative Commons Attribution 4.0 International license.

### Metabolism of phenolic compounds in the fermentation of pu-erh tea.

Phenolic compounds possess one or more aromatic rings with one or more hydroxyl groups and generally are categorized as phenolic acids, flavonoids, coumarins, and tannins (27). Phenolic compounds, primarily catechins, are the characteristic chemical component in teas and provide a number of health benefits, such as reducing the incidence of coronary heart disease, diabetes, and cancer (28). Moreover, the oxidation of catechins and the production of oxidation reaction products in fermentation are crucial to the quality of black tea (29). Therefore, understanding the metabolism of phenolic compounds is essential for the investigation of the process and quality control of tea.

In this metabolomics study, 144 phenolic compounds were identified, including catechin 3-*O*-gallate, gallocatechin, and quercetin (Data Set S3, sheet 7). After fermentation, the relative levels of 73 metabolites decreased, which was in accordance with the decreasing levels of polyphenols and catechins, including EGC, EC, EGCG, C, and ECG, as shown by the spectrophotometric or HPLC analyses. Thus, phenolic compounds were actively metabolized, and the majority of levels of phenolic compounds decreased during fermentation. The decreasing content of phenolic compounds is responsible for the transformation of taste from astringent to mellow.

Seventy-two identified phenolic compounds grouped as phenolic glycosides, including luteone 7-glucoside, and quercetin 3,7-diglucuronide. The majority of the phenolic glycosides showed decreasing concentrations, except cerarvensin, catechin 3′-glucoside, terbutaline-1-glucuronide, and pelargonidin 3-arabinoside (Data Set S3, sheet 6). As described above, phenolic glycosides were suggested to be degraded by GHs, such as glucosidase, glucuronidase, galactosidase, and rhamnosidase (Table S1).

**FIG 5:**
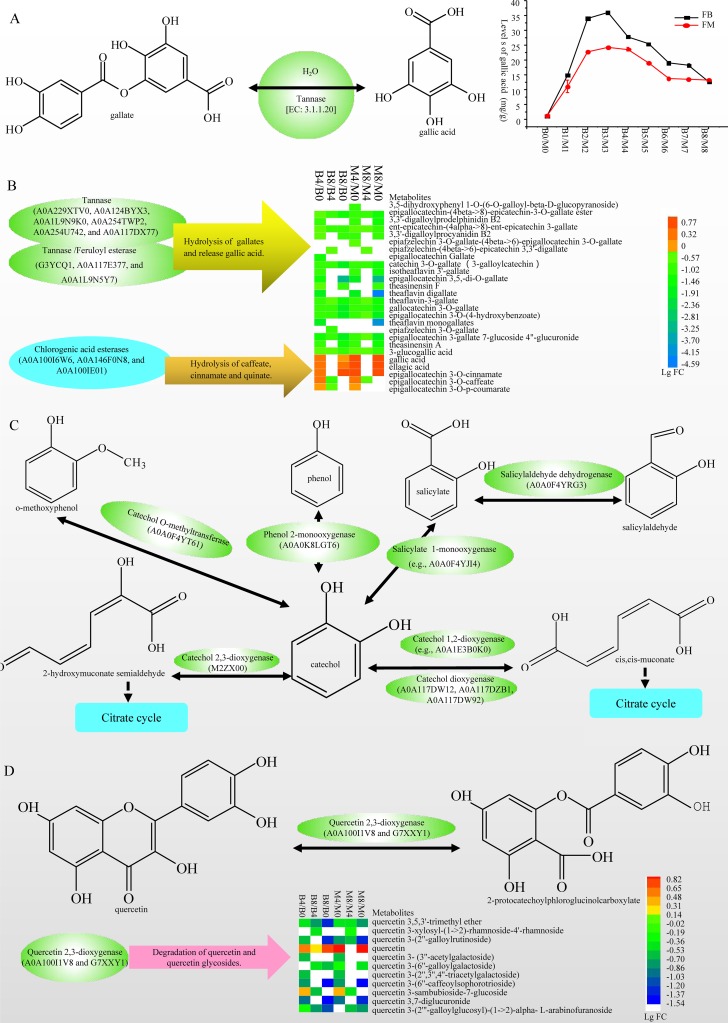
Enzymes involved in the metabolism of phenolic compounds. (A) Chemical reaction of tannase and the changes of gallic acid levels during fermentation. (B) Tannases, chlorogenate hydrolases, and the changing levels of gallates, caffeate, cinnamate, quinate, and gallic acid. (C) Enzymes and network for oxidation and degradation of catechol. (D) Chemical reaction of quercetin 2,3-dioxygenases and the changes in levels of quercetin and related compounds. Enzymes in panel C are shown in Table S2.

Twenty-one identified phenolic compounds were gallates, including EGCG, epiafzelechin 3-*O*-gallate, and theaflavin digallate, which showed decreasing contents during fermentation (Data Set S3, sheet 8). For example, the content of EGCG decreased from 47.3 ± 2.42 mg/g (B0) and 46.02 ± 2.81 mg/g (M0) to 0.08 ± 0.15 mg/g (B8) and was finally undetectable (M8). In contrast, the level of gallic acid increased more than 11 times (Table 2). Tannase (EC 3.1.1.20) catalyzes the breakdown of ester and depside linkages in hydrolyzable tannins, such as tannic acid, producing gallic acid and glucose (30) (Fig. 5A). In the metaproteomics analysis, six tannases (A0A229XTV0, A0A124BYX3, A0A1L9N9K0, A0A254TWP2, A0A254U742, and A0A117DX77) and three tannases/feruloyl esterases (G3YCQ1, A0A117E377, and A0A1L9N5Y7) were identified. Therefore, tannases were hypothesized to hydrolyze gallates and release gallic acid, which results in the changes of levels of these compounds in fermentation. Additionally, three chlorogenic acid esterases (EC 3.1.1.42) (A0A100I6W6, A0A146F0N8, and A0A100IE01) were identified, which were suggested to hydrolyze epigallocatechin 3-*O*-caffeate, epigallocatechin 3-*O*-*p*-coumarate, and epigallocatechin 3-*O*-cinnamate (Fig. 5B).

10.1128/mSystems.00680-19.6 TABLE S2 Enzymes involved in the metabolism of catechol-containing compounds. Download Table S2, DOCX file, 0.03 MB. Copyright © 2019 Zhao et al. 2019 Zhao et al. This content is distributed under the terms of the Creative Commons Attribution 4.0 International license.

Catechol is a phenol containing a benzene ring with adjacent hydroxyl groups. It can be considered the basic structural unit of phenolic compounds. According to the GO and KEGG annotations, we identified 19 enzymes (Table S2) involved in oxidation, conversion, and degradation of catechol and constructed a network as follows: catechol *O*-methyltransferase (A0A0F4YT61) catalyzes the formation of catechol from *o*-methoxyphenol, phenol 2-monooxygenase (A0A0K8LGT6) oxidizes phenols to form catechol, salicylaldehyde dehydrogenase (A0A0F4YRG3) oxidizes salicylaldehyde to produce salicylate, salicylate 1-monooxygenase (A0A0F4YJI4) oxidizes salicylate to form catechol, and catechol 2,3-dioxygenases (M2ZX00) and catechol 1,2-dioxygenase (A0A0K8LRX4, A0A117DW12, A0A117DZB1, and A0A117DW92) degrade catechol to form *cis*,*cis*-muconic acid or 2-hydroxymuconate semialdehyde (Fig. 5C). We hypothesized that aromatic rings or catechol in phenolic compounds was oxidized, modified, or degraded by these enzymes in fermentation. Additionally, quercetin 2,3-dioxygenase (A0A100I1V8), which catalyzes the decyclization of quercetin, was identified and suggested to degrade quercetin and quercetin glycosides (Fig. 5D).

We hypothesized a metabolic pathway of tea phenolic compounds resulting in the decrease of relative levels of most phenolic compounds and an increase in the content of several compounds including gallic acid, ellagic acid, quercetin, and myricetin in pu-erh fermentation as follows: (i) phenolic glycosides were hydrolyzed or synthesized by GHs and GTs; (ii) gallates were hydrolyzed by tannase and produced gallic acid; (iii) phenolic compounds were oxidized, modified, or degraded by catechol *O*-methyltransferase, phenol 2-monooxygenase, salicylaldehyde dehydrogenase, salicylate 1-monooxygenase, catechol 2,3-dioxygenases, catechol 1,2-dioxygenase, and quercetin 2,3-dioxygenase. To our knowledge, this is the first report on the enzymes involved in the metabolism of phenolic compounds in pu-erh tea fermentation, which are characteristic compounds in tea and are responsible for the taste and health benefits. Additionally, phenolic compounds are ubiquitously distributed phytochemicals found in most plant tissues and are important for the quality of plant-based foods (31); therefore, the findings in this article may provide interesting insight into other plant-based fermented foods and beverages.

### Development of the FFMP.

Fermented foods are important societal traditions and are crucial regional products in terms of the economy, as well as being rich in microbiological resources awaiting exploration (32). Understanding the microbiome within fermentation ecosystems is essential for maintaining traditional and artisanal practices in the context of urbanization, designing starter cultures, directing sensory quality, and improving the safety of the consumable products (6). Previously, Parente (33, 34) developed FoodMicrobionet, which provides a wealth of information on the structure of food biomes. We suggest developing the Food Fermentation Microbiome Project (FFMP) to study the microbiome within the food fermentation ecosystem using the powerful integration of meta-omics approaches. This work provides an example of a study of microbiomes in a fermented food ecosystem using integrated metabarcoding, metaproteomics, metabolomics, and HPLC approaches.

### Conclusion.

**FIG 6:**
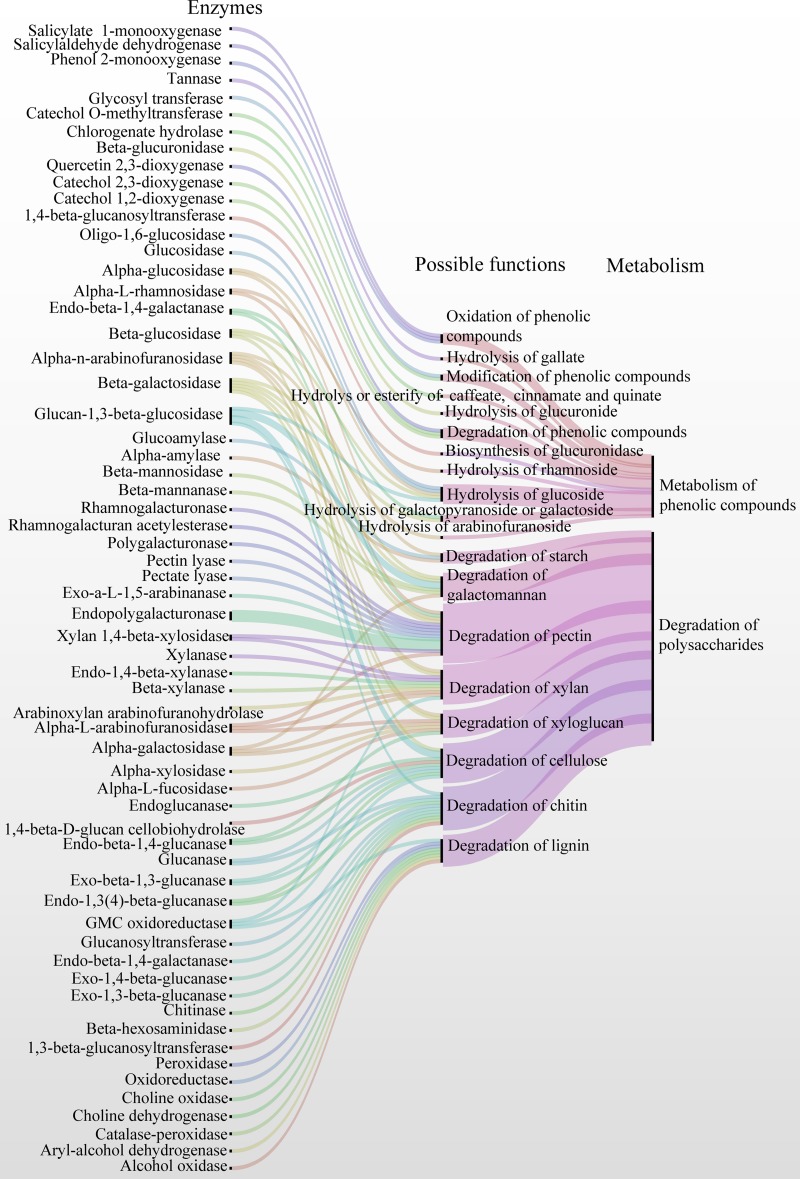
Overview of microbial enzymes involved in degradation of polysaccharides and metabolism of phenolic compounds.

Microbiomes in two fermentations of pu-erh tea were systematically examined via the integration of metabarcoding, metaproteomics, and metabolomics analyses. We identified the microbial succession and association, microbial activity, and changes in the metabolites during the fermentation of pu-erh tea. We found that microbiota produced CAZymes to degrade plant or fungal polysaccharides for their growth and reproduction, as well as enzymes involved in hydrolysis, oxidization, modification, or degradation of phenolic compounds (Fig. 6). This study advanced our understanding of the fermentation mechanism of pu-erh tea related to the microbial and metabolic succession, as well as the microbial functions during the fermentation of pu-erh tea.

## MATERIALS AND METHODS

### Fermentation of pu-erh tea and sample collection.

Two traditional fermentation processes of pu-erh tea were developed by the Yunnan D Tea Co., Ltd., Yunnan, China, between 10 October and 1 December 2014 (Table 1). Sun-dried green tea leaves purchased from Bajiazia Village (24°13′16.10″N, 98°24′0.18″E) and Mingzhi Mountain (24°09′33.74″N, 98°36′51.58″E), Mangshi City, Yunnan Province, China, were used as the raw material. The fermentation of pu-erh tea was developed according to the traditional method of spontaneous fermentation; raw materials, water, utensils, and the environment were not sterilized, and no starter was used. Based on the temperature of the tea piles and the experience of the manufacturer, the tea masses were broken down, washed with water to a moisture content of approximately 40%, mixed, and restacked in piles for 3 to 10 days. Samples of tea leaves were collected from the tea piles at five time points before each round of breaking up, mixing, and repiling. Samples of tea leaves were divided into two parts. One part was air dried and subjected to sensory evaluation, according to the protocol described by GB/T 23776-2009 (35), and analysis of the chemical compounds, while the second part was stored at −80°C. In total, 36 samples were collected and analyzed as outlined in Fig. 1 and Table 1. Detailed approaches are described in Text S1 in the supplemental material.

10.1128/mSystems.00680-19.1 TEXT S1 Detailed materials and methods. Download Text S1, DOCX file, 0.04 MB. Copyright © 2019 Zhao et al. 2019 Zhao et al. This content is distributed under the terms of the Creative Commons Attribution 4.0 International license.

### Metabarcoding of bacterial 16S rRNA gene and fungal ITS sequence.

To analyze the taxonomic composition of the bacterial and fungal communities, the universal primer pairs 515F (5′-GTGCCAGCMGCCGCGGTAA-3′) and 907R (5′-CCGTCAATTCMTTTRAGTTT-3′) and ITS1F (5′-CTTGGTCATTTAGAGGAAGTAA-3′) and ITS1R (5′-GCTGCGTTCTTCATCGATGC-3′), which incorporate Illumina adapters and barcode sequences, were used to amplify the V4-V5 hypervariable region of bacterial 16S rRNA genes, as well as the ITS1 of fungal 18S rRNA genes using a two-step amplification procedure. DNA extraction, PCR, and Illumina MiSeq sequencing (2- by 150-bp reads) were performed by TinyGene Technology Co., Ltd. (Shanghai, China). Each sample was extracted for two replicates, and each extraction was analyzed twice. Analysis of OTU cooccurrence networks was developed using the CoNet application (36) on Cytoscape 3.7.1 (37). The detailed approaches are described in Text S1 in the supplemental material.

### Metaproteomics experiments.

The microbial proteins in tea leaves were extracted by Tris-HCl–phenol and methanol precipitation, measured using the Bradford method with bovine serum albumin as a standard, and validated with sodium dodecyl sulfate-polyacrylamide electrophoresis (as described in our previous report [19]). For each sample of tea leaves, three independent extractions were carried out. A total of 200 μg of protein was digested with trypsin according to the filter-aided sample preparation protocol (38). Liquid chromatography-tandem mass spectrometry (LC-MS/MS) analysis of each replicate of peptide extracts was performed using an Easy-nLC1000 coupled to a QExactivePlus mass spectrometer (Thermo Fisher Scientific, Bremen, Germany). Raw data were processed using Thermo Proteome Discoverer software version 1.4 (Thermo Fisher Scientific, Bremen, Germany) with the default settings. The MS/MS data were queried against the UniProt database (http://www.uniprot.org/) with the following search parameters: carbamidomethylation of cysteine as the fixed modification, oxidation of methionine and deamidation of glutamine and asparagine as variable modifications, a maximum of two missed cleavages, a precursor ion mass tolerance of 10 ppm, and an MS/MS tolerance of 0.05 Da. Decoy database searches were performed with a false-discovery rate (FDR) cutoff of 1%. GO annotations for the identified proteins were assigned according to those reported in the UniProt database. COG annotations of identified proteins were computed using eggNOG-Mapper based on eggNOG 4.5 orthology data (39, 40). The CAZymes annotation was developed by dbCAN (41). The KEGG pathway was annotated using the KEGG (Kyoto Encyclopedia of Genes and Genomes) Automated Annotation Server (KAAS) using the bidirectional best hit BLAST method (https://www.genome.jp/tools/kaas/) (42). Detailed approaches are described in Text S1 in the supplemental material.

### Metabolomics experiments.

Metabolites were extracted from the raw material (B0-1, B0-2, M0-1, and M0-2), the fourth repiling (B4-1, B4-2, M4-1, and M4-2), and the final fermented tea leaves (B8-1, B8-2, M8-1, and M8-2) and assessed using a UPLC-quadrupole time of flight (Q-TOF) MS-based metabolomics approach. The tea powders (50 mg) mixed with 20 μl of l-2Cl-Phe (0.03 mg/ml) as internal standard were extracted with 1 ml of 70% methanol for 30 min in an ultrasonic bath. The extraction was kept at −20°C for 20 min. Then, the samples were centrifuged at 14,000 rpm at 4°C for 10 min, and 200 μl of supernatant was filtered using 0.2-μm polytetrafluoroethylene filters and subjected to UPLC-Q-TOF–MS analysis at Majorbio Bio-Pharm Technology Co., Ltd. (Shanghai, China). Triplicate preparations and analyses were performed for each sample. Detailed approaches are described in Text S1 in the supplemental material.

### Analysis of chemical compounds in tea leaves by HPLC and spectrophotometry.

The contents of water extractions (WEs), tea polyphenols (TPs), free amino acids (FAA), and soluble sugar (SS) in tea leaves were analyzed using the spectrophotometric method described by Wang et al. (24). The amount of gallic acid, caffeine, hydrolyzable tannins (1,4,6-tri-*O-*galloyl-β-d-glucose [GG]), and catechins, including (+)-catechin (C), (−)-epicatechin (EC), (−)-epigallocatechin (EGC), (−)-epicatechin 3-*O*-gallate (ECG), and (−)-epigallocatechin 3-*O*-gallate (EGCG), in the tea leaves was determined using HPLC with an Agilent 1200 series HPLC system (Agilent Technologies, Santa Clara, CA, USA), as described in our previous study (19). Three replicates of each sample were extracted, and each extraction was detected twice. Data were analyzed and statistical analyses were performed in SPSS 19.0. Significant differences between two groups were noted by different letters (*P* < 0.05). Detailed approaches are described in Text S1 in the supplemental material.

### Data availability.

The sequencing data of bacterial 16S rRNA genes and the fungal ITS1 of 18S rRNA gene are available at the Sequence Read Archive under project code SRP139059. The mass spectrometry proteomics data have been deposited in the ProteomeXchange Consortium (http://proteomecentral.proteomexchange.org) via the iProX partner repository (43) with the identifier PXD012223.
