# Supplementary figures and images for: Integrated Meta-omics Approaches To Understand the Microbiome of Spontaneous Fermentation of Traditional Chinese Pu-erh Tea
Source: mSystems. 2019 Nov 19;4(6):e00680-19. doi: 10.1128/mSystems.00680-19 (PMC6867877; doi:10.1128/mSystems.00680-19)

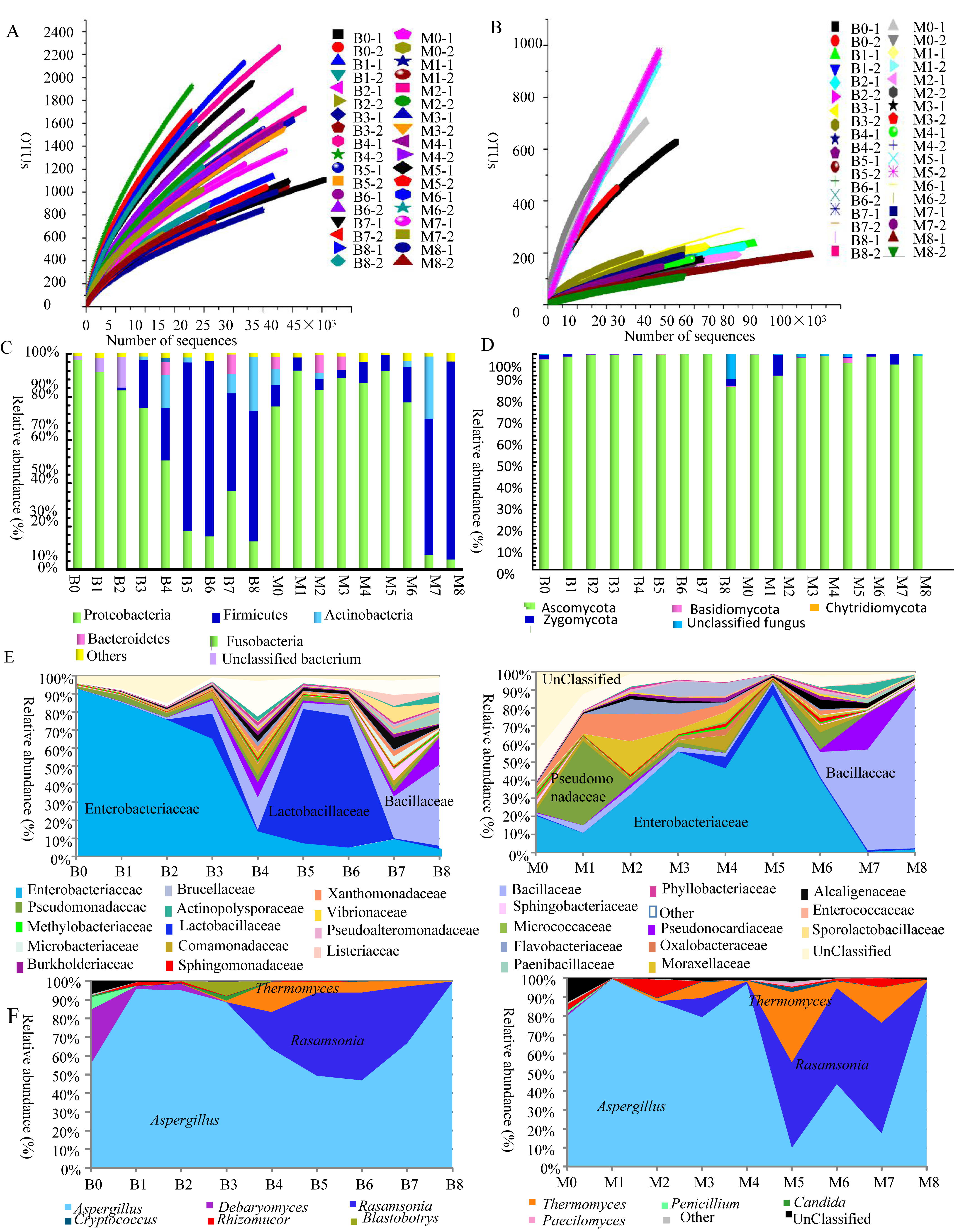

Supplement: FIG S1 [file mSystems.00680-19-sf001.tif]

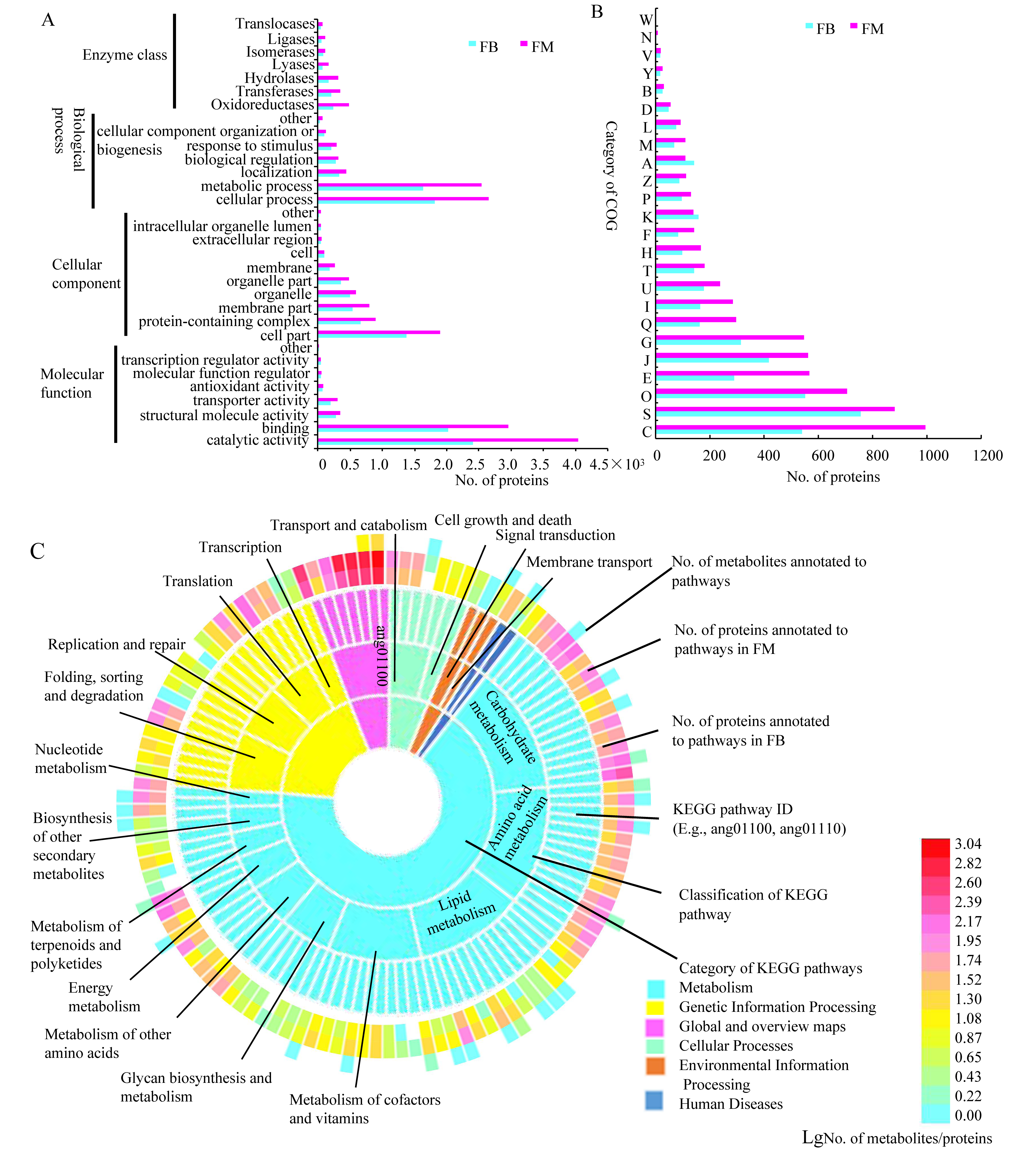

Supplement: FIG S2 [file mSystems.00680-19-sf002.tif]

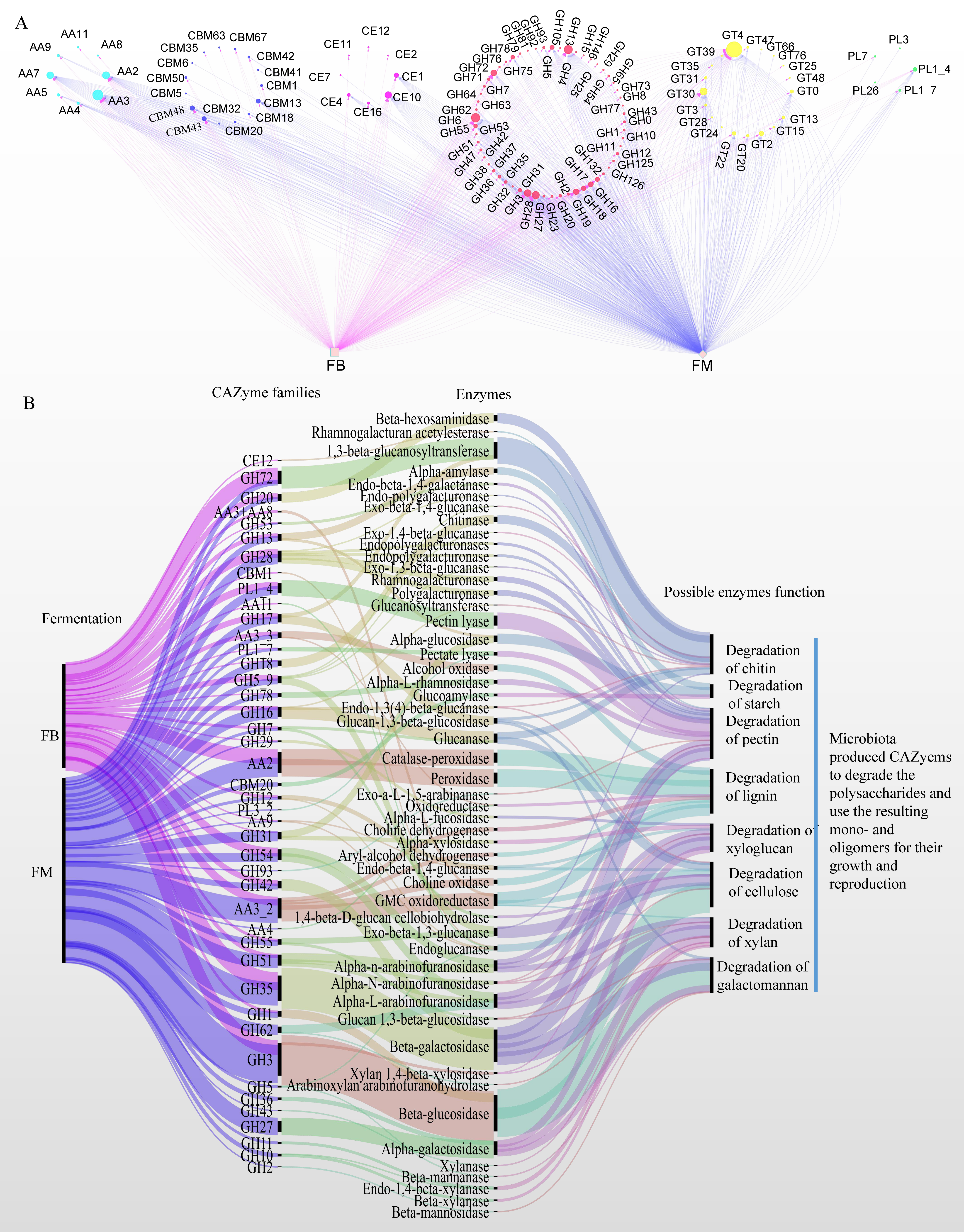

Supplement: FIG S3 [file mSystems.00680-19-sf003.tif]
