# Supplementary material for: Integrated Meta-omics Approaches To Understand the Microbiome of Spontaneous Fermentation of Traditional Chinese Pu-erh Tea
Source: mSystems. 2019 Nov 19;4(6):e00680-19. doi: 10.1128/mSystems.00680-19 (PMC6867877; doi:10.1128/mSystems.00680-19)
Supplement: TABLE S1 [file mSystems.00680-19-st001.docx]

**TABLE S1** Enzymes involved in hydrolysis of glycosides

| Uniprot-AC | Glycosidase | Organism | Cazyems family | Possible function |
| --- | --- | --- | --- | --- |
| A0A146G2E8 | alpha-n-arabinofuranosidase | *A. flavus* | GH51 | hydrolysis of arabinofuranoside |
| A0A146FUW8 | alpha-n-arabinofuranosidase | *A. luchuensis* | GH54+CBM42 |  |
| A0A1M3TBD5 | arabinogalactan endo-beta-1,4-galactanase | *A. luchuensis* | GH53 | hydrolysis of galactopyranoside or galactoside |
| A0A0F4Z133 | beta-galactosidase | *A. wentii* | GH35 |  |
| A0A0L1J957 | beta-galactosidase | *A. ruber* | GH35 |  |
| A0A1J6WUZ7 | beta-galactosidase | *A. brasiliensis* | GH42 |  |
| A0A1L9RJP3 | beta-galactosidase | *A. niger* | GH35 |  |
| A0A2G7G438 | alpha-glucosidase | *A. niger* | GH13+GT25 | hydrolysis of glucoside |
| A0A1F2K440 | beta-glucosidase | *A. niger* | GH1 |  |
| A0A1M7GMT3 | beta-glucosidase | *A. niger* | GH3 |  |
| S3XPC1 | beta-glucosidase | *A. bombycis* | GH3 |  |
| A0A076V327 | beta-glucosidase | *A. nomius* | GH3 |  |
| A0A0F4YZ53 | beta-glucosidase | *Microbacterium sp.* | GH3 |  |
| A0A0L1J5B3 | beta-glucosidase | *A. niger* | GH3 |  |
| A0A117E2E5 | beta-glucosidase | *Flavobacterium pectinovorum* | GH3 |  |
| A0A1M3TAS7 | beta-glucosidase | *Propionibacterium sp.* | GH3 |  |
| A0A0F4YXA2 | glucan-1,3-beta-glucosidase | *A. luchuensis* | GH17 |  |
| A0A100IGU3 | mannosyl-oligosaccharide glucosidase | *A. oryzae* | GH63 |  |
| A0A124BY47 | neutral alpha-glucosidase ab | *R.emersonii* | GH31 |  |
| A0A100IRZ6 | oligo-1,6-glucosidase | *A. niger* | GH13_40 |  |
| A0A146FD64 | oligo-1,6-glucosidase | *A. niger* | GH13_40 |  |
| A0A100IUA9 | beta-glucuronidase | *A. brasiliensis* | GH79 | hydrolysis of glucuronide |
| A0A0F4YK02 | 1,3-beta-glucanosyltransferase | *A. tubingensis* | GH72 | biosynthesis of glucuronidase |
| A0A0R1I3B8 | alpha-L-rhamnosidase | *A. luchuensis* | GH78 | hydrolysis of rhamnoside |
