# Supplementary material for: Integrated Meta-omics Approaches To Understand the Microbiome of Spontaneous Fermentation of Traditional Chinese Pu-erh Tea
Source: mSystems. 2019 Nov 19;4(6):e00680-19. doi: 10.1128/mSystems.00680-19 (PMC6867877; doi:10.1128/mSystems.00680-19)
Supplement: TABLE S2 [file mSystems.00680-19-st002.docx]

**TABLE S2** Enzymes involved in metabolism of catechol-containing compounds

| Uniprot-AC | Protein names | Organism | GO (biological process) | GO (molecular function) |
| --- | --- | --- | --- | --- |
| A0A0F0I3C6 | 12-HQD like protein | *A. parasiticus* | catechol-containing compound metabolic process [GO:0009712] | catechol 1,2-dioxygenase activity [GO:0018576]; ferric iron binding [GO:0008199] |
| A0A2J5I462 | Aromatic compound dioxygenase | *A. taichungensis* | [GO:0009712] | [GO:0018576]; [GO:0008199] |
| A0A0K8LRX4 | Catechol 1,2-dioxygenase | *A. udagawae* | [GO:0009712] | [GO:0018576]; [GO:0008199]; transferase activity, transferring phosphorus-containing groups [GO:0016772] |
| A0A117DW12 | Catechol 1,2-dioxygenase | *A. niger* | [GO:0009712] | [GO:0018576]; [GO:0008199] |
| A0A117DZB1 | Catechol 1,2-dioxygenase | *A. niger* | [GO:0009712] | [GO:0018576]; [GO:0008199] |
| A0A117DW92 | Catechol 1,2-dioxygenase | *A. niger* | [GO:0009712] | [GO:0018576]; [GO:0008199] |
| A0A117DWV6 | Dioxygenase | *A. niger* | [GO:0009712] | [GO:0018576]; [GO:0008199] |
| Q6MY62 | Dioxygenase | *A. fumigatus* | [GO:0009712] | [GO:0018576]; [GO:0008199] |
| A0A0F4YJI0 | Hydroxyquinol 1,2-dioxygenase | *R.emersonii* | [GO:0009712] | [GO:0018576]; [GO:0008199]; hydroxyquinol 1,2-dioxygenase activity [GO:0018581] |
| A0A0F4YLZ4 | Uncharacterized protein | *R. emersonii* | [GO:0009712] | [GO:0018576]; [GO:0008199] |
| A0A0F4YLM7 | Uncharacterized protein | *R. emersonii* | [GO:0009712] | [GO:0018576]; [GO:0008199] |
| A0A1E3B0K0 | Uncharacterized protein | *A. cristatus* | [GO:0009712] | [GO:0018576]; [GO:0008199] |
| A0A1H9YKC1 | 2-keto-4-pentenoate hydratase/2-oxohepta-3-ene-1,7-dioic acid hydratase (Catechol pathway) | *Nonomuraea wenchangensis* |  | catalytic activity [GO:0003824] |
| M2ZX00 | Catechol 2,3-dioxygenase | *Rhodococcus ruber* |  | dioxygenase activity [GO:0051213] |
| A0A0F4YT61 | Catechol O-methyltransferase | *R. emersonii* |  | O-methyltransferase activity [GO:0008171] |
| A0A0K8LGT6 | Phenol 2-monooxygenase | *A. udagawae* | spliceosomal snRNP assembly [GO:0000387] | FAD binding [GO:0071949]; monooxygenase activity [GO:0004497] |
| A0A0F4YRG3 | Salicylaldehyde dehydrogenase | *R. emersonii* |  | salicylaldehyde dehydrogenase activity [GO:0018485] |
| A0A0F4YJI4 | Salicylate 1-monooxygenase | *R. emersonii* |  | [GO:0071949]; salicylate 1-monooxygenase activity [GO:0018658] |
| A0A100I1V8 | Quercetin 2,3-dioxygenase | *A. niger* |  | dioxygenase activity [GO:0051213] |
