# Supplementary material for: Integrated Meta-omics Approaches To Understand the Microbiome of Spontaneous Fermentation of Traditional Chinese Pu-erh Tea
Source: mSystems. 2019 Nov 19;4(6):e00680-19. doi: 10.1128/mSystems.00680-19 (PMC6867877; doi:10.1128/mSystems.00680-19)
Supplement: TEXT S1 [file mSystems.00680-19-s0001.docx]

**Fermentation of pu-erh tea and sample** **collection.** Two traditional fermentations of pu-erh tea were developed by the Yunnan D Tea Co., Ltd, Yunnan, China, between October 10 and December 1, 2014. Sun-dried green tea leaves used as the raw material were purchased from Bajiazia Village (N 24°13’16.10” N 98°24’0.18” E) and Mingzhi Mountain (24°09’33.74” N 98°36’51.58” E), Mangshi City, Yunnan Province, China. The fermentation of pu-erh tea was developed according to the traditional method of spontaneous fermentation; raw materials, water, utensils and environment were not sterilized, and no starter was used. Each fermentation contained approximately 10 metric tonnes of raw material. The sun-dried tea leaves were showered with water to a moisture content of ~40% (as judged based on the experience of the manufacturer). The tea mass was mixed thoroughly during the showering process. The wet tea mass was then stacked in piles one meter high and covered with moist gunny cloth to maintain its moisture content and temperature during fermentation. Based on the temperature of the tea piles and the experience of the manufacturer, the tea masses were broken down, showered with water to a moisture content of approximately 40%, mixed and re-stacked in piles for 3-10 days. Samples of tea leaves were collected from the tea piles at five time points before each round of breaking up, mixing and re-piling. Samples of tea leaves were divided into two parts. One part was air-dried and subjected to sensory evaluation, according to the protocol described by GB/T 23776-2009(1) (Fig. 1), and analysis of the chemical compounds, while the second part was stored at -80°C. When the fermented tea leaves were reddish-brown and the taste was no longer astringent, the tea piles were broken down, the tea leaves were air-dried to a water content of approximately 10% and the fermentation process was stopped. In total, 36 samples were collected and analyzed, as outlined in Fig. 1 and Table 1.

**Meta-barcoding of bacterial 16S rRNA gene and fungal ITS sequence.** DNA extraction, PCR and Illumina MiSeq sequencing (2 × 150 bp reads) were performed by TinyGene Technology Co., Ltd. (Shanghai, China). Microbial genomic DNA was extracted and purified from each of the collected samples using the CTAB methods as following: Tea leaves (5 g) were suspended in 50 mL of 0.9% NaCl buffer and vibrated in a rotary shaker at 200 rpm for 30 min. The extractions were filtered using 2-layer of gauze and the tea leaves were discarded. The supernatants were centrifuged at 10,000 rpm at for 10 min. The precipitates were resuspended in 3 mL of 0.9% NaCl buffer and 800 μL of resuspension were centrifuged at 10,000 rpm for 5 min. The precipitates were rinsed with 500 μL of 0.9% NaCl buffer twice. The precipitates were resuspended in 1 mL of CTAB extraction buffer containing 100 mM Tris-HCl (pH 8.0), 20 mM EDTA (pH 8.0), 2% CTAB and 1.4 M NaCl, supplemented with 500 μL 20% SDS, 30.8 μL proteinase K (10 mg/mL), 3.8 μL 10 mg ribonuclease A (10 mg/mL), 100 μL lysozyme (50 mg/mL) and 100 μL β-mercaptoethanol. The mixture was incubated at 55 °C for 2 h with occasional vortexing. The mixture was extracted with 1000 μL of phenol/chloroform/isoamyl-alcohol (25/24/1) and centrifuged at 10,000 rpm for 10 min. The supernatant was then incubated at -20 °C with 0.1 -fold of NaAC (3 M, pH 5.0) and 0.6-fold of cold isopropanol for overnight. And centrifuged at 12,000 rpm for 10 min. The precipitate was rinsed with 500 μL 70% ethanol twice and then dissolved in 40 μL TE buffer. 3 μL of DNA sample was electrophoresed on 1.2% agarose gel. The isolated DNA was stored at -80 °C until the PCR stage. Each sample was extracted two replicates, and each extraction was analyzed twice.

To analyze the taxonomic composition of the bacterial and fungal community, the universal primers [pair](javascript:void(0);)s of 515F (GTGCCAGCMGCCGCGGTAA) (2) with 907R (CCGTCAATTCMTTTRAGTTT) (3), and ITS1F (5′-CTTGGTCATTTAGAGGAAGTAA-3′) (4) with ITS1R (5′- GCTGCGTTCTTCATCGATGC -3′) (5), which incorporate Illumina adapters and barcode sequences, were used to amplify the V4-V5 hypervariable region of bacterial 16S rRNA genes and the internal transcribed spacer region 1 (ITS1) of fungal 18S rRNA genes using a two-step amplification procedure. The first step reaction mix contained 1×buffer, 0.2 mM dNTPs, 0.2 μM of the primers, 20-50 ng genomic DNA, and 1 unit of Phusion HiFiTaq polymerase (NEB) in a volume of 50 μL. The amplification program consisted of an initial denaturation step at 94 °C for 2 min. This was followed by 30 cycles, where a cycle consisted of 94 °C for 30 s (denaturation), 56 °C for 90 s (annealing), and 72 °C for 30 s (extension), followed by a final extension of 72 °C for 2 min. The PCR reactions were purified using AxyPrep PCR Clean-up Kit (Axygen Biosciences, CA, USA) according to the manufacturer’s protocol. 5 μl of the eluate served as template for the second step. Reaction components were the same as in the first step except that the amount of Taq was reduced to 0.8 units and a reaction volume of 40 μL was used. The entire 40 μL volume was amplified as above for eight cycles. PCR products were purified as above and were then quantified using a Qubit 2.0 Fluorometer (Invitrogen) with the Qubit dsDNA HS Assay Kit (Invitrogen, Carlsbad, CA, USA). Integrity of the DNA was confirmed by electrophoresis in a 1.2 % agarose gel with 1 × TAE buffer.

Libraries were sequenced on an Illumina MiSeq platform (2 × 300 bp reads) at TinyGene Technology Co., Ltd., Shanghai, China. Data preprocessing was performed mainly using Mothur V.1.33.3 software (6, 7). Sequences with any ambiguous bases, sequences with more than 8 homopolymers and sequences with lengths less than 200 bp were removed using the screen. seqs command in Mothur. Putative chimeric sequences were detected and removed via the Chimera Uchime algorithm contained within Mothur. Sequences with similarities > 97% were clustered into one operational taxonomic unit (OTU). The taxonomical assignment of each OTU was performed using the classify.seqs command (Naïve Bayesian Classifier) against the SILVA 16S rRNA gene database (release 119) (8-10) in Mothur at an 80% cutoff with 1000 iterations. Community richness and diversity indices [Chao1 estimator, abundance-based coverage estimator (ACE) and Shannon indices, respectively] and rarefaction curves were obtained using Mothur (11). Principal components analysis (PCA) was performed on the relative abundance matrix of OTU data of each sample in Canoco 5.0 (Biometrics, Wageningen, Netherlands) (12).The sequencing data generated for this study were submitted to the Sequence Read Archive (SRA) and are available under project of SRP139059.

**Metaproteomics experiments.** The microbial proteins in each sample of tea leaves was extracted by Tris-HCl/Phenol and Methanol precipitation, measured by the Bradford method using bovine serum albumin as a standard, and validated by SDS-PAGE described in our previous report (13). For each tea leaves sample, three independent extractions were performed. 200μg of proteins was digested with trypsin according to the Filter Aided Sample Preparation (FASP) protocol (14). LC-MS/MS analyses of the peptide extracts were performed using an Easy-nLC1000 coupled to a QExactivePlus mass spectrometer (Thermo Fisher Scientific, Bremen, Germany) for each sample replicate. Lyophilized peptides were reconstituted in 14 μL of 2% acetonitrile and 0.05% trifluoroacetic acid, and an aliquot of 5 μL (approximatively 7 μg total peptide) was loaded onto a Nano Trap Column (C18, 3 μm, 100 Å, 75 μm×150 mm) fitted with a Nano Trap guard column (C18, 5 μm, 100 Å, 100 μm × 20 mm) (Thermo Fisher Scientific, Bremen, Germany). The mobile phase consisted of water and acetonitrile containing 0.1% formic acid (v/v). The LC gradient was set as follows: 0-2 min, 3-5% B; 2-34 min, 5-19% B; 34-38 min, 19- 90% B; 38-50 min, solvent B was kept at 90%, and then dropping it to 0% for another 20 min; the flow rate was 300 nL/min. MS data were acquired in a data-dependent mode. The spray voltage was set to 2.1 kV, the heated capillary temperature was 280 °C. Survey full scan MS spectra (from m/z 350 to 1600) were acquired with a resolution of 70,000 and an AGC (Automatic Gain Control) target value of 3 × 10^6^ ions. The 50 most intense precursors were selected for higher-energy collisional dissociation (HCD) MS/MS (resolution, 17,500; NCE, 27; AGC at 2e5; maximum fill time, 50 ms).

Raw data were processed using Thermo Proteome Discoverer software version 1.4 (Thermo Fisher Scientific, Bremen, Germany) with the default settings. The MS/MS data were queried against the UniProt database (http://www.uniprot.org/) with the following search parameters: carbamidomethylation of cysteine as the fixed modification, oxidation of methionine and deamidation of glutamine and asparagine as variable modifications, a maximum of two missed cleavages, a precursor ion mass tolerance of 10 ppm and an MS/MS tolerance of 0.05 Da. Decoy database searches were performed with a false discovery rate (FDR) cutoff of 1%. Gene Ontology (GO) annotations for the identified proteins were assigned according to those reported in the UniProt database. Cluster of Orthologous Groups (COG) annotations of identified proteins were computed using eggnog-mapper (15) based on eggNOG 4.5 orthology data (16). The Carbohydrate-active enzyme Annotation was developed by dbCAN (17). KEGG analysis of the unique-proteins identified in fermentation B and M were annotated using the KEGG (Kyoto Encyclopedia of Genes and Genomes) Automated Annotation Server (KAAS) using the bi-directional best hit BLAST method (https://www.genome.jp/tools/kaas/) (18).

**Metabolomics experiments.** To monitor the dynamics of metabolome in fermentation, the metabolomes in raw materials (B0-1, B0-2, M0-1 and M0-2), samples of 4^th^ re-piling (B4-1, B4-2, M4-1 and M4-2) and final fermented tea leaves (B8-1, B8-2, M8-1 and M8-2) were extracted and analyzed by UPLC-MS/MS based Metabolomics approach. Triplicate preparations and analyses were performed for each sample. The Metabolomics analysis was performed at Majorbio Bio-Pharm Technology Co., Ltd., Shanghai, China. For UPLC-Q-TOF/MS, L-2Cl-Phe was added as an internal standard. The tea powers (50 mg) mixed with 20 μL of L-2Cl-Phe (0.03 mg/mL) were extracted with 1 mL of 70% methanol for 30 min in ultrasonic bath. And the extraction was kept at -20 °C for 20 min. Then, the samples were centrifuged at 14,000 rpm at 4 °C for 10 min, and 200 μL supernatants were filtered using 0.2 μm polytetrafluoroethylene (PTFE) filters and subjected to UPLC-Q-TOF/MS analysis. UPLC-Q-TOF/MS analysis was performed using a UPLC Acquity system™ equipped with a C18 reversed-phase column (100 × 2.1 mm, 1.7 μm, Acquity BEH, Waters, Milford, USA). The column temperature was maintained at 45 °C. The mobile phase consisted of water and acetonitrile containing 0.1% formic acid (v/v). The solvent gradient was set as follows: 0-2 min, 5-20 % B; 2-8 min, 5-35 min, 20-60 % B; 8-12 min, 60-100 % B; 12-14 min, held at 100 % B; 14-14.5 min, 100-5 % B; 14.5-15.5 min holding at 5 % B. The flow rate was maintained at 0.4 mL/min and 3 μL of sample was injected. The mass spectrometric data was collected using a Waters VION IMS Q-TOF Mass Spectrometer equipped with an electrospray ionization (ESI) source operating in either positive or negative ion mode. The source temperature and desolvation temperature was set at 120 ℃ and 500 ℃, respectively, with a desolvation gas flow of 900 L/h. The capillary voltage, cone capillary and collision energy were 1.0 kV, 40V and 6 eV, respectively. Centroid data was collected from 50 to 1,000 m/z with a scan time of 0.1 s and interscan delay of 0.02 s over a 13 min analysis time. The UPLC-Q-TOF/MS raw data were analyzed by Progenesis QI Informatics (Waters Corporation, Milford, USA) for peak alignment, peak picking and data normalisation. Peak picking thresholds were set between 0.5 and 14.0 min; mass range 50-1,000 Da, mass tolerance 0.01 Da. There were 5993 and 5430 detectable peaks in UPLC-Q-TOF/MS positive and negative mode, and combined 11424 m/z. The combined three-dimensional matrix, including m/z, peak RT and peak intensities, and RT-m/z pairs were introduced into the SIMCA-P+ 14.0 software package (Umetrics, Umeå, Sweden) for multivariate statistical analysis. PCA and orthogonal partial least squares discriminant analysis (OPLS-DA) were carried out after mean centering and unit variance scaling to visualize differences in the metabolomes of the experimental groups. Variable importance in the projection (VIP) was used to rank the overall contribution of each variable to the OPLS-DA model, and those variables with VIP > 1.0, *p* < 0.05 and fold change (FC) > 1.5 were classified as differentially changed metabolites (DCMs). DCMs were characterized using the METLIN database ([metlin.scripps.edu](http://metlin.scripps.edu/)) (19, 20). Pathway analysis was performed using MetaboAnalyst 3.0 (http://www.metaboanalyst.ca/) (21). DEMs were grouped according to the classifications in The Human Metabolome Database (http://www.hmdb.ca/) (22), the LIPID MAPS Structure Database (http://www.lipidmaps.org/data/structure/index.php) (23).

**Analysis of chemical compounds in tea leaves by HPLC and spectrophotometry.** The contents of water extractions, tea polyphenols, free amino acids (FAA), and polysaccharides in tea leaves were analyzed using the spectrophotometric method described by Wang et al. (24). The amount of gallic acid, caffeine, hydrolyzable tannins (1,4,6-tri-*O-*galloyl-*β*-D-glucose [GG]), and catechins, including (+)-catechin (C), (−)-epicatechin (EC), (−)-epigallocatechin (EGC), (−)-epicatechin 3-*O*-gallate (ECG), and (−)-epigallocatechin 3-*O*-gallate (EGCG) in the tea leaves was determined using HPLC with an Agilent 1200 series HPLC system (Agilent Technologies, Santa Clara, CA), as described in our previous work (13).

The levels of luteolin, myricetin, quercetin, taxifolin, kaempferol, ellagic acid in tea leaves was determined by HPLC using an Agilent 1200 series. The tea powers (1.0 g) were extracted with 40 mL methanol and 4 mL Hydrochloric acid by the refluxing method in water bath at 85 °C for 90 min. And the extraction was filtered through absorbent cotton and made up to 50 mL by adding methanol. 1000 μL supernatants were filtered using 0.45 μm polytetrafluoroethylene (PTFE) filters and subjected to HPLC analysis. The separation was completed using an Poroshell 120 EC- C18 column (4.6× 100 mm, 2.7 μm, Agilent Technologies, Santa Clara, CA) fitted with a UPLC Guard (Poroshell 120 EC- C18, 4.6× 5 mm, 2.7 μm, Agilent Technologies, Santa Clara, CA). The mobile phases were solvents A (0.2% phosphoric acid) and B (80% methanol). Elution conditions were as follows: 0-0.2 min, solvent A was reduced from 100 to 85% and solvent B from 0 to 15% (linear gradient); 0.2-13 min, solvent B was increased to 28% (linear gradient); 13-20 min, solvent B was increased to 50% (linear gradient); 20-33.50 min solvent B was increased to 80% (linear gradient); 34.0 min solvent B was increased to 100% and kept at 100% for 6 min; 40.0 min solvent A was increased from 0 to 100% (linear gradient); 40.0-50.0 min solvent A was kept at 100%; the flow rate was 0.6 mL/min. The temperature of the column oven was set at 40 C. The Ultraviolet detector was set at 265 nm.

Each sample was extracted three replicates, and each extraction was detected twice. Data were analyzed and statistics performed in SPSS 19.0. Significant differences between two groups were noted by asterisks (*p* <0.05).

**References**

1. Gong, S., L. Chengyin, L. Xu, Z. Yuxiang, S. Hong, G. Yalin, W. Jihong, Z. Lei, and G. Zilei. 2009. Methodology of sensory evaluation of tea GB/T 23776-2009. In, China Agriculture Press. 2009.
2. Sylvain, F. E., B. Cheaib, M. Llewellyn, C. T. Gabriel, F. D. Barros, V. A. Luis, and N. Derome. 2016. pH drop impacts differentially skin and gut microbiota of the Amazonian fish tambaqui (Colossoma macropomum). Sci Rep 6:32032.
3. Ishii, K., M. Fukui, and S. Takii. 2000. Microbial succession during a composting process as evaluated by denaturing gradient gel electrophoresis analysis. J Appl Microbiol 89:768-77.
4. Gardes, M., and T. D. Bruns. 1993. ITS primers with enhanced specificity for basidiomycetes--application to the identification of mycorrhizae and rusts. Mol Ecol 2:113-8.
5. White, T. J., T. Bruns, S. Lee, and J. W. Taylor. 1990. Amplification and direct sequencing of fungal ribosomal RNA genes for phylogenetics. PCR protocols: a guide to methods and applications 18:315-322.
6. Schloss, P. D., D. Gevers, and S. L. Westcott. 2011. Reducing the effects of PCR amplification and sequencing artifacts on 16S rRNA-based studies. PLoS One 6:e27310.
7. Schloss, P. D., S. L. Westcott, T. Ryabin, J. R. Hall, M. Hartmann, E. B. Hollister, R. A. Lesniewski, B. B. Oakley, D. H. Parks, C. J. Robinson, and Others. 2009. Introducing mothur: open-source, platform-independent, community-supported software for describing and comparing microbial communities. Applied and environmental microbiology 75:7537--7541.
8. Huse, S. M., L. Dethlefsen, J. A. Huber, W. D. Mark, D. A. Relman, and M. L. Sogin. 2008. Exploring microbial diversity and taxonomy using SSU rRNA hypervariable tag sequencing. PLoS Genet 4:e1000255.
9. Quast, C., E. Pruesse, P. Yilmaz, J. Gerken, T. Schweer, P. Yarza, J. Peplies, and F. O. Glockner. 2013. The SILVA ribosomal RNA gene database project: improved data processing and web-based tools. Nucleic Acids Res 41:D590-6.
10. Wang, Q., G. M. Garrity, J. M. Tiedje, and J. R. Cole. 2007. Naive Bayesian classifier for rapid assignment of rRNA sequences into the new bacterial taxonomy. Appl Environ Microbiol 73:5261-7.
11. Kemp, P. F., and J. Y. Aller. 2004. Bacterial diversity in aquatic and other environments: what 16S rDNA libraries can tell us. FEMS Microbiol Ecol 47:161-77.
12. Šmilauer, P., and J. Lepš. 2014. Multivariate Analysis of Ecological Data using CANOCO 5. Bulletin of the Ecological Society of America 86:201-201.
13. Zhao, M., D. L. Zhang, X. Q. Su, S. M. Duan, J. Q. Wan, W. X. Yuan, B. Y. Liu, Y. Ma, and Y. H. Pan. 2015. An Integrated Metagenomics/Metaproteomics Investigation of the Microbial Communities and Enzymes in Solid-state Fermentation of Pu-erh tea. Sci Rep 5:10117.
14. Wisniewski, J. R., A. Zougman, N. Nagaraj, and M. Mann. 2009. Universal sample preparation method for proteome analysis. Nat Methods 6:359-62.
15. Huerta-Cepas, J., K. Forslund, L. P. Coelho, D. Szklarczyk, L. J. Jensen, C. von Mering, and P. Bork. 2017. Fast Genome-Wide Functional Annotation through Orthology Assignment by eggNOG-Mapper. Mol Biol Evol 34:2115-2122.
16. Huerta-Cepas, J., D. Szklarczyk, K. Forslund, H. Cook, D. Heller, M. C. Walter, T. Rattei, D. R. Mende, S. Sunagawa, M. Kuhn, L. J. Jensen, C. von Mering, and P. Bork. 2016. eggNOG 4.5: a hierarchical orthology framework with improved functional annotations for eukaryotic, prokaryotic and viral sequences. Nucleic Acids Res 44:D286-93.
17. Yin, Y., X. Mao, J. Yang, X. Chen, F. Mao, and Y. Xu. 2012. dbCAN: a web resource for automated carbohydrate-active enzyme annotation. Nucleic Acids Res 40:W445-51.
18. 18. Moriya, Y., M. Itoh, S. Okuda, A. C. Yoshizawa, and M. Kanehisa. 2007. KAAS: an automatic genome annotation and pathway reconstruction server. Nucleic Acids Res 35:W182-5.
19. Zhu, Z. J., A. W. Schultz, J. Wang, C. H. Johnson, S. M. Yannone, G. J. Patti, and G. Siuzdak. 2013. Liquid chromatography quadrupole time-of-flight mass spectrometry characterization of metabolites guided by the METLIN database. Nat Protoc 8:451-60.
20. Smith, C. A., G. O'Maille, E. J. Want, C. Qin, S. A. Trauger, T. R. Brandon, D. E. Custodio, R. Abagyan, and G. Siuzdak. 2005. METLIN: a metabolite mass spectral database. Ther Drug Monit 27:747-51.
21. Xia, J., I. V. Sinelnikov, B. Han, and D. S. Wishart. 2015. MetaboAnalyst 3.0--making metabolomics more meaningful. Nucleic Acids Res 43:W251-7.
22. Wishart, D. S., Y. D. Feunang, A. Marcu, A. C. Guo, K. Liang, R. VázquezFresno, T. Sajed, D. Johnson, C. Li, and N. Karu. 2018. HMDB 4.0: the human metabolome database for 2018. Nucleic Acids Research 46.
23. Sud, M., E. Fahy, D. Cotter, A. Brown, E. A. Dennis, C. K. Glass, J. Alfred H. Merrill, R. C. Murphy, C. R. H. Raetz, and D. W. Russell. 2007. LMSD: LIPID MAPS structure database. Nucleic Acids Research 35:527-32.
24. Wang, Q., C. Peng, and J. Gong. 2011. Effects of enzymatic action on the formation of theabrownin during solid state fermentation of Pu-erh tea. J Sci Food Agric 91:2412-8.
